# Monitoring viral evolution and epidemiological characteristics of SARS-CoV-2 during 2022–2023 using Integrated Genomic Surveillance

**DOI:** 10.1038/s43856-026-01647-x

**Published:** 2026-05-26

**Authors:** Christin Mache, Romy Kerber, Jessica Schulze, Marie Lataretu, Susan Abunijela, Yusra Seyam, Sofia Paraskevopoulou, Djin-Ye Oh, Maximilian Arlt, Aleksandar Radonić, Somayyeh Sedaghatjoo, Matthias Budt, Ann-Sophie Lehfeld, Felix Hartkopf, Ralf Dürrwald, Torsten Semmler, Walter Haas, Stephan Fuchs, Stefan Kröger, Thorsten Wolff, Integrated Molecular Surveillance for SARS-CoV-2 (IMSSC2) Laboratory Network, Gerald von Hermanni, Felix Stelter, Laura Gropp, Bernhard Miller, Patrick Chhatwal, Christina Kiel, Abdullah Al Mamun, Konrad A. Bode, Veronika Balau, Anja Kruggel, Roger Grosser, Aida Bajraktarevic, Ralf Ignatius, Juliane Flindt, Ingo Neumann, Manuel Haffner, Jana Löbner, Karsten Mydlak, Andi Krumbholz, Thomas Lorentz, Semir Doric, Thomas Müller, Denise Buhlmann, Beate Hermann, Claudia Gerlich, Andreas Gerritzen, Carsten Tiemann, Udo Geipel, Bettina Georg, Meike Voss, Sandra Dehn, Friedemann Tewald, Patrick Finzer, Lea Klar, Florian Szabados, Michael Erren, Jerzy Roch Nofer, Alexa Laubner, Markus Petzold, Annika Brinkmann, Andreas Nitsche, Christin Mache, Jessica Schulze

**Affiliations:** 1 Influenza and other Respiratory Viruses (Unit 17), Robert Koch Institute, Berlin, Germany; 2 Respiratory Infections (Unit 36), Robert Koch Institute, Berlin, Germany; 3 Genome Competence Centre (MF1), Robert Koch Institute, Berlin, Germany; 4 Labor Augsburg MVZ GmbH, August-Wessels-Straße 5, Augsburg, Germany; 5 MVZ Labor PD Dr. Volkmann und Kollegen SE & Co. GbR, Gerwigstraße 67, Karlsruhe, Germany; 6 MVZ Labor Limbach Lehrte, Auf den Pohläckern 12, Lehrte, Germany; 7 MVZ Labor Dessau GmbH, Bauhüttenstrasse 6, Dessau-Roßlau, Germany; 8 MVZ Labor Dr. Limbach & Kollegen eGbR, Im Breitspiel 16, Heidelberg, Germany; 9 IMD - MVZ Labor Greifswald GmbH, Vitus-Bering- Straße 27a, Greifswald, Germany; 10 Labor Dr. Wisplinghoff, Horbeller Str. 18-20, Köln, Germany; 11 MVZ Labor 28 GMBH, Mecklenburgische Straße 28, Berlin, Germany; 12 Labor MVZ Westmecklenburg, Ellerried 7, Schwerin, Germany; 13 MVZ GANZIMMUN GmbH, Erich-Dombrowski-Straße 3, Mainz, Germany; 14 MVZ Gemeinschaftslabor Cottbus, Uhlandstraße 53, Cottbus, Germany; 15 Labor Dr. Krause & Kollegen MVZ GmbH, Steenbeker Weg 23, Kiel, Germany; 16 SYNLAB MVZ Weiden GmbH, Zur Kesselschmiede 4, Weiden, Germany; 17 MVZ DIANOVIS GmbH, Straße des Friedens 2, Gera, Germany; 18 MVZ Medizinisches Labor Bremen GmbH, Bremen, Germany; 19 MVZ Labor Krone GbR, Siemensstraße 40, Bad Salzuflen, Germany; 20 Bioscientia MVZ Labor Saar GmbH, Otto-Kaiser-Straße 8a, St. Ingbert, Germany; 21 MVZ Labor Ravensburg SE & Co. eGbR, Ravensburg, Germany; 22 Labor Prof. Dr. G. Enders MVZ GbR, Rosenbergstraße 85, Stuttgart, Germany; 23 MVZ Düsseldorf-Centrum GbR, Immermannstrasse 65A, Düsseldorf, Germany; 24 Laborarztpraxis Osnabrück, Rostockerstrasse 5-7, Georgsmarienhütte, Germany; 25 MVZ Laborzentrum Weser-Ems, Bischofsstr. 1, Osnabrück, Germany; 26 Institute for Medical Microbiology and Virology, University Hospital Carl Gustav Carus, TUD Dresden University of Technology, Fetscherstraße 74, Dresden, Germany; 27 Highly Pathogenic Viruses (ZBS 1), Robert Koch Institute, Berlin, Germany

**Keywords:** SARS-CoV-2, Viral infection

## Abstract

**Background:**

The continued emergence of SARS-CoV-2 variants with increased transmissibility and immunoevasive properties highlights the necessity to complement genomic surveillance with epidemiological data and laboratory-based assessment of viral phenotypes. Effective surveillance tools must be scalable, cost-efficient, and able to detect and characterize emerging variants in timely manner.

**Methods:**

We utilized the Integrated Molecular Surveillance for SARS-CoV-2 (IMSSC2) network to conduct nationwide integrated SARS-CoV-2 genomic surveillance in Germany. SARS-CoV-2-positive samples from diagnostic laboratories were routinely subjected to whole genome sequencing. Epidemiological data from COVID-19 cases infected with BA.1, BA.2, BA.5.1, BQ.1.1, XBB.1.5, XBB.1.9.1, and XBB.1.9.2, notified between 1 December 2021 and 30 April 2023, were analyzed. Complementary, representative virus isolates were examined for immunoevasive properties and replication in human respiratory infection models.

**Results:**

Lineage assignments of 4595 SARS-CoV-2 genomes indicate ongoing viral evolution with successive replacement of dominant variants from Delta to Omicron lineages BA.1, BA.2, BA.5.1, BQ.1, and XBB recombinants. Age-stratified epidemiological analyses show higher proportions of BA.1 and BA.2 infections in children. Multivariable logistic regression identifies male sex and increasing age as significant predictors of hospitalization. Phenotypic characterization suggests ongoing adaptation of Omicron variants BA.2, BQ.1.1, XBB.1.5, and XBB.1.9.2 to the upper human respiratory tract and enhanced propagation of XBB.1.9.2 in an alveolar infection model.

**Conclusion:**

Integrated genomic, epidemiological and virological approaches enable early assessment of emerging SARS-CoV-2 lineages and demonstrate ongoing adaptation across the human respiratory tract. Our findings show that geographically representative, scalable surveillance provides robust insights into viral evolution, supporting sustainable surveillance beyond the acute pandemic phase.

## Abstract (web summary)

Mache, Kerber, et al. conduct nationwide integrated genomic, epidemiological, and virological surveillance of SARS-CoV-2 variants in Germany. They identify sequential variant replacements, age-specific infection patterns, clinical risk factors, and phenotypic evidence of continued adaptation to the human respiratory tract.

## Plain language summary

SARS-CoV-2 continues to change and produce new variants that may spread more easily or escape population immunity. For risk assessment, it is important to combine genetic analyses of the virus with information about infected cases and laboratory experiments to assess how the virus has changed. Using the nationwide surveillance network for Integrated Molecular Surveillance of SARS-CoV-2 (IMSSC2), we monitored the distribution of Omicron variants between December 2021 and April 2023. We found that various variants became dominant, and some appeared more common in children. Older people and men had a higher risk of hospitalization when infected with Omicron. Laboratory experiments showed continued adaptation of variants to the human respiratory tract. Together, our findings provide a framework for early assessment of emerging variants.

## Introduction

SARS-CoV-2 has been evolving in humans since its introduction into the human population in 2019^1^. This led to the transient dominance of phenotypically adapted variant groups, such as Alpha, Delta, and Omicron. Those variants were characterized by increased transmissibility and/or mutations in viral genes conferring escape from population immunity, thereby driving waves of COVID-19 with substantial morbidity and mortality. Within weeks after its initial emergence in November 2021 in southern African countries, Omicron reached global predominance^2,3^. It featured more than 30 amino acid changes in the antigenic spike protein compared to previous variants and had an elevated capability to evade population immunity^2,4,5^. Omicron’s continuous evolution creates an expanding range of descendant lineages, including the recombinant BA.2-derived XBB lineages that began circulating in October 2022^6^.

During the acute phase of the pandemic, in late 2020, Germany substantially expanded SARS-CoV-2 sequencing through a statutory mandate (Coronavirus Surveillance Verordnung - CorSurV) requiring the submission of genomic data from diagnostic laboratories to a central repository (Deutscher Elektronischer Sequenzdaten-Hub (DESH)), with financial reimbursement provided^7–9^. The acute phase of the global response to the COVID-19 pandemic ended on May 5, 2023, when the World Health Organization (WHO) formally lifted the Public Health Emergency of International Concern status^10^. However, the WHO and the European Centre for Disease Prevention and Control (ECDC) have recommended to continue genomic surveillance of SARS-CoV-2 beyond its acute phase in order to protect public health in the post-pandemic phase, as viral variants with resurgent virulence may arise in the future^11^. While the CorSurV generated vast amounts of full-genome SARS-CoV-2 sequences, it was resource-intensive and challenging to sustain long-term. Thus, SARS-CoV-2 surveillance that is both informative and long-term cost-effective is essential. To this end, in Germany, the nationwide network of laboratories (Integrated Molecular Surveillance of SARS-CoV-2 (IMSSC2) laboratory network) was launched in November 2020, which operated via centralized sequencing and genome reconstruction at the Robert Koch Institute (RKI)^12^. Since then, it has been the only nationwide genomic surveillance system for SARS-CoV-2 variants in Germany that is continuously operational all year round until today.

For effective disease surveillance, we set out to complement virus genomic data provided by DESH and through the IMSSC2 laboratory network in a timely manner with epidemiological data provided by local health authorities within mandatory notification. Generally, such an “Integrated Genomic Surveillance” (IGS) approach should enable the identification of concerning variants early after their emergence and gather essential information on their epidemiological growth (as a proxy for transmissibility), their ability to cause severe disease in different age groups, and specific virological characteristics.

A variant’s effective transmissibility and virulence in a population are determined by its intrinsic transmissibility and pathogenicity, as well as the host population’s immunity. A variety of approaches — including epidemiological analyses, mathematical prediction models, and laboratory-based investigations employing animal models or complex models of the human respiratory tract — can be used to estimate the risk potential of viral variants^13,14^. Over the course of the pandemic, immunologically naïve populations have become practically nonexistent; consequently, epidemiological and clinical data now reflect the virus’ effective, rather than intrinsic, transmissibility and pathogenicity. For an assessment of these parameters, genomic analyses are complemented by laboratory-based experimental characterization of a new variant’s virological phenotype. To this end, infection models of the human upper respiratory tract (URT) can serve as a proxy to estimate for a given viral variant the extent of shedding into infectious respiratory particles, which is a prerequisite for spread within a population. As a complementary approach, growth phenotype analyses in models of the distal human lung may mirror the pathogenic potential and virulence of the respective variant in naïve individuals. Additionally, serum neutralization experiments are to date the fastest way to inform about a new variant’s immunoevasive properties.

Here, we report on analyses of 4595 SARS-CoV-2 genomes collected through the IMSSC2 surveillance network in Germany between December 2021 and the end of the acute pandemic phase in April 2023. Lineage distributions changed over time, reflecting the consecutive replacements of dominant variants from Delta to the Omicron lineages BA.1, BA.2, BA.5, and BQ.1 to XBB recombinants. As a first step towards public health risk assessment, 84,639 COVID-19 cases infected with BA.1, BA.2, BA.5.1, BQ.1.1, XBB.1.5, XBB.1.9.1, and XBB.1.9.2, which were notified to the German mandatory surveillance system, were used in epidemiological analyses. In parallel, we conducted experimental evaluations of predominant Omicron sublineages, assessing their adaptation to efficient replication in physiologically relevant systems representative of the human respiratory tract, to support the interpretation of epidemiological observations.

## Methods

At the RKI, we developed the IMSSC2 laboratory network, a geographically balanced nationwide laboratory network comprising 24 primary diagnostic laboratories from 14 of 16 federal states of Germany. Laboratories contributing to the SARS-CoV-2 genomic surveillance submit five randomly selected SARS-CoV-2-positive samples per week throughout the entire observation period. This number of samples corresponds to the minimum requirements for detecting SARS-CoV-2 lineages with a prevalence of 3% in the pool of all circulating variants, given an incidence of 50 cases per 100,000 people. SARS-CoV-2-positive patient materials are sent to the RKI for genome sequencing and phylogenetic analyses. Prototypic viruses are isolated from swab material via passaging in human Caco-2 cells and phenotypically assessed through replication analyses on human respiratory epithelial cell culture systems differentiated at the air-liquid-interface (ALI).

### Sample Selection and RNA extraction

Samples of SARS-CoV-2-positive patient’s material were selected as described previously^12^. In brief, IMSSC2 network laboratories routinely test samples from outpatient health centers and hospitals. For representative sampling, IMSSC2 network laboratories randomly select SARS-CoV-2 samples each week that meet the following criteria: (i) different zip codes to capture geographically diverse cases and minimize the likelihood of samples being collected from the same cluster, (ii) Ct values below 23, which are associated with more accurate whole genome sequencing (WGS) results. To ensure complete traceability of the samples, the sample labels and shipping material are delivered to the network laboratories in advance (before shipment) by the RKI.

For the extraction of total RNA from URT specimens (either nasal, nasopharyngeal or oropharyngeal swabs) at RKI, the Magna Pure 96 DNA and Viral RNA Small Volume kit (Roche Life Science, Mannheim, Germany) and the Magna Pure instrument (Roche Life Science, Mannheim, Germany) were used according to the manufacturer’s instructions. For approximately 10% of the sequenced samples, RNA extractions were already performed on-site at network laboratories, and extracted RNA was sent to RKI.

### Sequencing and genome reconstruction

Nanopore libraries for SARS-CoV-2 sequencing were prepared using the NEBNext® ARTIC SARS-CoV-2 Companion kit (New England Biolabs, Frankfurt am Main, Germany) according to the manufacturer’s protocol employing Mosquito HV Genomics and Dragonfly (SPT Labtech, Melbourne, UK) liquid handling instruments. We utilized the ARTIC V4 to V5.3.2 primer sets for amplicon generations (see date, sets and primer sequences provided in Zenodo^15^). Barcoding of the samples was performed using the Native Barcoding Expansion kit (EXP-NBD196) and the Ligation Sequencing kit (SQK-LSK109) from Oxford Nanopore Technology. The prepared libraries, consisting of 24 to 96 samples, were pooled and loaded into a R9.4.1 flow cell and from 20.01.2023 onward on R10.4.1 for 12 to 24 h, depending on the number of samples per run, resulting in an average of 116k reads per sample, which could be attributed to SARS-CoV-2. Consensus genomes were then timely reconstructed with most current poreCov version available at the respective time^16^ with basecalled FASTQ files. In brief, the read quality is summarized by NanoPlot^17^ and taxonomical classification by Kraken2^18^ and krona^19^. The raw FASTQ files are filtered by length and genomes are reconstructed by the ARTIC pipeline (https://github.com/artic-network/fieldbioinformatics) using medaka as variant caller (https://github.com/nanoporetech/medaka). Thereafter, the lineage of the reconstructed genomes is assigned with pangolin (https://github.com/cov-lineages/pangolin), mutations are analyzed with Nextclade^20^ and the genome quality is assessed by PRESIDENT.

### Curation and quality control of genomic sequences

#### IMSSC2 laboratory network dataset

To prepare the IMSSC2 dataset, all samples collected in the IMSSC2 laboratory network between December 1st, 2021, and April 30th, 2023, were selected. The final dataset contained a total number of 4595 randomly sampled sequences with valid quality control (QC) criteria. We re-ran poreCov v1.9.2 in FASTA-mode with ‘--n_threshold 0.2‘ on the final data set (*n* = 4595 sequences) for a uniform QC output and lineage assignment. For sequence QC, poreCov uses PRESIDENT (v0.6.8) (https://github.com/rki-mf1/president) to compare the reconstructed sequences to the Wuhan reference sequence (NC_045512.2) using a sequence similarity threshold of 90% and allowing up to 20% N bases (‘--n_threshold 0.2‘). The N content across the IMSSC2 dataset has a median of 1.47% (0.42% min., 1.75% mean, 9.71% max.). The overall IMSSC2 dataset sequence identity to the Wuhan reference has a median of 98.01% (89.90% min., 97.83% mean, 99.42% max.). PANGO lineages were assigned using pangolin version 4.3 with pangolin-data version 1.23.1 (https://github.com/cov-lineages/pangolin)^21^.

#### DESH dataset

The DESH dataset contains the nationwide collection of all samples sequenced in Germany during the pandemic’s acute phase and in compliance with the CorSurV. Reconstructed full-genome consensus sequences were submitted to the central collection platform DESH. The RKI published the DESH sequences on GitHub and Zenodo^9^. Duplicates of full-genome sequences were rejected during submission based on the transaction ID, submitting lab, sampling date, FASTA sequence, and header. All QC-filtered and randomly collected sequences only with a sampling date between December 1st, 2021, and April 30th, 2023, were extracted. The resulting dataset contained *n* = 511,533 sequences. Lineages were assigned as described above.

### Phylogenetic tree inference

Genomes that belong to the randomly sampled IMSSC2 dataset (*n* = 4595) were aligned with MAFFT v7.490 using default parameters^22^. Phylogenetic inference was performed with IQ-TREE v2.2.0.3^23^ under the GTR + F + R2 evolutionary model, using 1000 ultra-fast bootstrap replicates^24^, and the resulting tree was visualized and colored in Iroki^25^. Phylogenetic analysis revealed five long branch attractions, which were subsequently removed with *treeshrink* v1.3.9 using default parameters^26^.

### Analysis of COVID-19 case epidemiological data

**Fig. 1:**
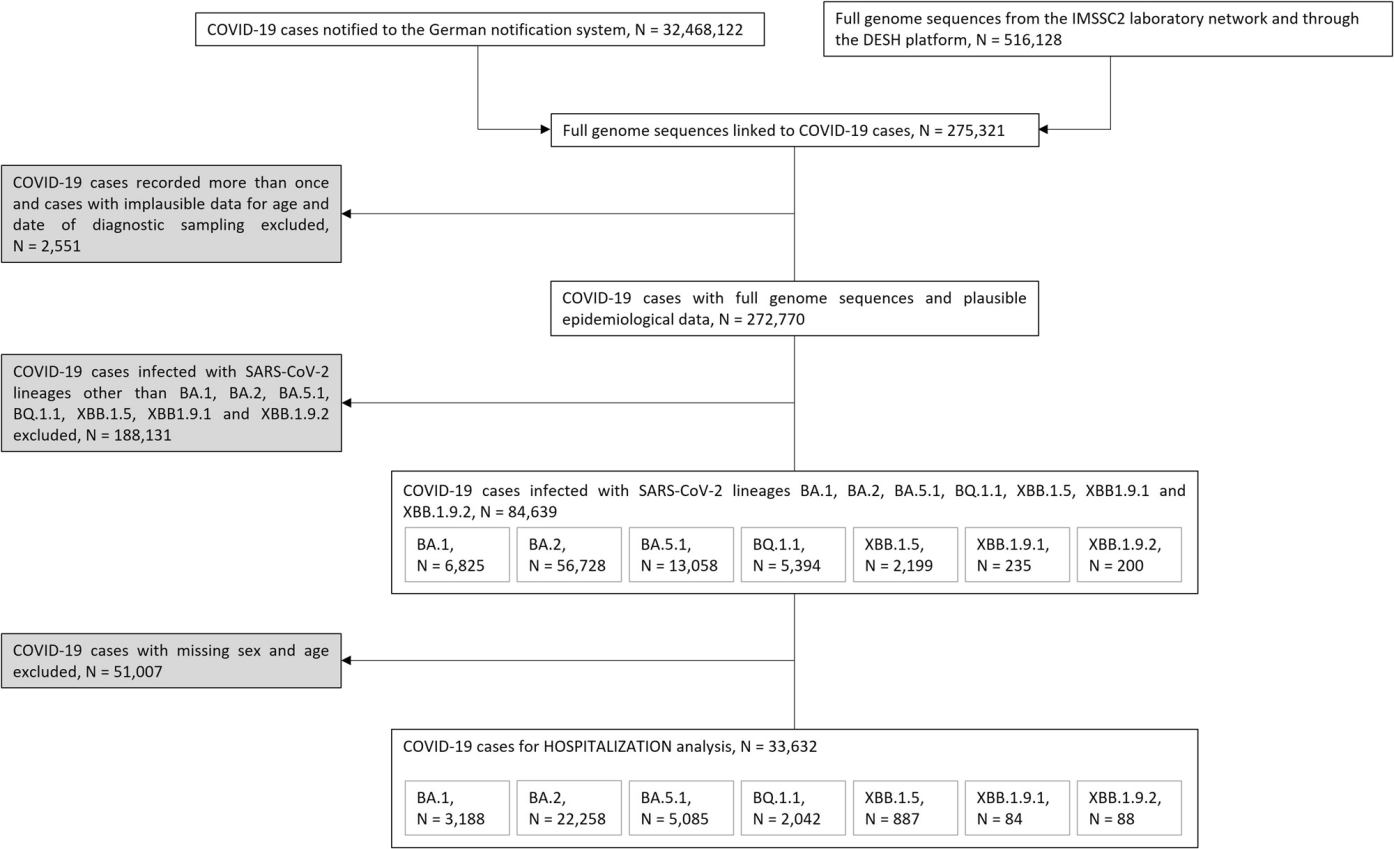
Selection of COVID-19 cases. Flow Chart of the Selection of COVID-19 Cases included in the Lineage-specific Analysis (N = 84,639) and in the Sub-Analysis on Hospitalization (N = 33,632) in Germany, December 1st, 2021, to April 30th, 2023.

Epidemiological data of COVID-19 cases with virus genomic information were retrospectively analyzed. Data for laboratory-confirmed COVID-19 cases were provided through the mandatory German national surveillance system by public health authorities. From December 1st, 2021, to April 30th, 2023, 32,468,122 COVID-19 cases were notified to the national surveillance (Fig. 1 and Table S1).

As of December 9th, 2023, 516,128 genome sequences of SARS-CoV-2 lineages were available, originating from both the IMSSC2 laboratory network and DESH, and were submitted as randomly selected samples. The integrated dataset was generated by merging consensus SARS-CoV-2 sequences, assigned PANGO lineages, and epidemiological data on the case level using a unique identifier provided in the data from the German national surveillance system and in the metadata of SARS-CoV-2 genomes. This unique identifier was also utilized to deduplicate COVID-19 cases recorded more than once. Additionally, COVID-19 cases with implausible data for age and sampling date were excluded. The resulting cleaned integrated database contained 272,770 COVID-19 cases (Fig. 1 and Table S1). Cases were further selected for retrospective analysis based on inclusion/exclusion criteria outlined in Fig. 1. First, only cases infected with the most prevalent SARS-CoV-2 variants over a continuous period of at least 8 weeks in Germany were included. Based on this criterion, individuals infected with lineages BA.2, BA.5.1, BQ.1.1, and recombinant lineage XBB.1.5 were selected. In order to provide a comprehensive description of the reporting period, cases infected with BA.1, as the earliest Omicron lineage, as well as cases infected with XBB.1.9.1 and XBB.1.9.2, classified as Variants under Monitoring (VUM) by the WHO in spring 2023, were included in the analysis. Sublineages of the aforementioned lineages and other SARS-CoV-2 variants occurring during the study period were excluded. The final dataset comprised 84,639 COVID-19 cases (Fig. 1). To investigate the distribution of infections among different age groups, individuals were categorized into six age groups: 0–4, 5–14, 15–34, 35–59, 60–79, and 80 years and older. Second, to analyze the association between hospitalization and independent variables using logistic regression, a subset of data was generated, by excluding COVID-19 cases with missing information on hospitalization status, sex, age, and month of diagnostic sampling. The final hospitalization dataset comprised 33,632 COVID-19 cases (Fig. 1).

### Cell culture

For cultivation of Caco-2 (ATCC HTB-37) and Vero E6 (ATCC CRL-1586) cells, Dulbecco’s Modified Eagle Medium (DMEM, Thermo Fisher Scientific, Darmstadt, Germany, 52100) containing 10% fetal bovine serum (FBS, Merck, Darmstadt, Germany, F7524) supplemented with 2mM L-glutamine (Carl Roth, Karlsruhe, Germany, HN08.3), 100 U/ml penicillin, 100 μg/ml streptomycin (PAN-Biotech, Aidenbach, Germany, P06-07100), 1x non-essential amino acids (Carl Roth, Karlsruhe, Germany, 9185.1), and 1 mM sodium pyruvate (Carl Roth, Karlsruhe, Germany, 9182.1) was used.

Human Alveolar Epithelial Lentivirus immortalized (hAELVi, inSCREENex, Braunschweig, Germany, INS-CI-1015) cells were cultivated in huAEC Medium (inSCREENex, Braunschweig, Germany, INS-ME-1013-500 ml). Prior to application of the cells, the culture flasks were coated with huAEC Coating solution (inSCREENex, Braunschweig, Germany, INS-SU-1018-100 ml). For polarization, approximately 9 × 10^5^ cells were seeded into the apical chamber of a pre-coated filter insert. Cells were initially incubated under liquid-liquid-conditions for 3 days, followed by cultivation under ALI conditions for up to 28 days. For infection experiments, polarized hAELVi cells were used following incubation under ALI conditions for at least 21 days.

Reconstructed human nasal and bronchial epithelium cultures were obtained from Epithelix (Plan-les-Ouates Switzerland, MucilAir^TM^ EP01MD) and further cultivated in MucilAir™ culture medium (Epithelix, Plan-les-Ouates, Switzerland, EP05MM) under ALI conditions upon arrival, according to the manufacturer’s instructions. Nasal and bronchial cultures were derived from 3 healthy single donors each. All cells were incubated in a humidified atmosphere at 37 °C with 5% CO_2_.

### Isolation of SARS-CoV-2 viruses from patient samples

For isolation of primary SARS-CoV-2 isolates, selected samples were sterile filtered (0.2 μm) and subsequently used to inoculate approximately 2 × 10^5^ Caco-2 cells after the presence of a particular virus lineage had been determined by WGS. After incubation at 37 °C and 5% CO_2_ for 72 h, the supernatant was harvested and used for high-titer stock production. For preparation of virus stocks, approximately 1 × 10^7^ Caco-2 cells were infected at a multiplicity of infection (MOI) of 0.001 and incubated at 37 °C and 5% CO_2_ for 48 h. After incubation, cell debris was removed by centrifugation, and aliquots of stock solution were stored at -80 °C. The absence of second-site mutations was confirmed by WGS. Virus isolation and subsequent assays with infectious material were performed under biosafety level (BSL) 3 conditions at the RKI, Berlin.

### Infection of human respiratory cells and virus titration on Vero E6 cells

Cells were infected with SARS-CoV-2 D614G (hCoV-19/Germany/BW-RKI-N-0001/2020, GISAID accession: EPI_ISL_481253), SARS-CoV-2 Delta B.1.617.2 (ENA project PRJEB50616; sequence ID IMSSC2-206-2021-00148), SARS-CoV-2 Omicron BA.2 (ENA project PRJEB55524; accession ID ERS12788649), SARS-CoV-2 Omicron BA.5.1 (GISAID accession: EPI_ISL_14419656), SARS-CoV-2 Omicron BQ.1.1 (GISAID accession: EPI_ISL_16883461), SARS-CoV-2 Omicron XBB.1.5 (GISAID accession: EPI_ISL_18530775), SARS-CoV-2 Omicron XBB.1.9.1 (GISAID accession: EPI_ISL_17006863), or SARS-CoV-2 Omicron XBB.1.9.2 (GISAID accession: EPI_ISL_17069408), respectively.

Cells were washed once with PBS (Vero E6) or D-PBS (ALI human cell cultures) and then inoculated with virus diluted in D-PBS/0.3% BA. For ALI cultures, the virus solution was applied to the apical chamber of the filter insert. After incubation for 1 h at 37 °C, cells were washed twice with PBS or D-PBS, as appropriate, and fresh medium was added to the cells. For ALI cultures, medium was added to the basolateral compartment of the filter insert.

To perform replication analysis, supernatants were harvested at indicated time points and stored at -80 °C until titration by standard Plaque Assay on Vero E6 cells to quantify infectious virus particles. For replication analysis on Vero E6 cells, 10% of the supernatant was harvested and refilled with fresh culture medium. To collect samples of ALI cultures, 50 μl (MucilAir^TM^) or 250 μl D-PBS (hAELVi), was used for apical washes at 37 °C for 30 min. The increase of viral titers during the early infection phase was calculated using linear regression between 0 and 16 h post infection (p.i.) from replication analyses.

### Plaque reduction neutralization test

Plaque reduction neutralization test (PRNT) was performed as described previously^12^. Briefly, 1.6 ×10^5^ Vero E6 cells were plated in 24-well plates the day before. WHO reference serum panels NIBSC 21/338 (a pool of 265 SARS-CoV-2 seropositive donors) or NIBSC 20/142 (a pool of SARS-CoV-2 negative human plasma), respectively, were 2-fold serially diluted and incubated with 50 PFU of SARS-CoV-2 isolates in a total volume of 200 μl for 1 h at 37 °C. The mixture was then used to infect the cells for 1 h at 37 °C. After aspiration of the inoculate, cells were grown for three days in Avicel plaque medium and stained with crystal violet. The PRNT50 titer represents the reciprocal value of the highest serum dilution that reduces plaque number by at least 50% compared to untreated infection.

### Cytokine ELISA

Basolateral supernatants of infected cells were used to quantify immune activation. Samples were analyzed according to the manufacturer’s instructions using the Human IFN-beta DuoSet ELISA Kit (R&D Systems Inc., Minneapolis, USA, DY814) and the Human IL-29/IL-28B (IFN-lambda 1/3) DuoSet ELISA Kit (R&D Systems Inc., Minneapolis, USA, DY1598B).

### Statistics and reproducibility

Statistical analyses of epidemiological data were performed using R version 4.3.0^27^. Categorical variables are presented as numbers and percentages of patients. Percentages were calculated based on all observations, including missing values for data completeness. To assess the distribution of infections across sex, age groups, hospitalized cases, mortality, and vaccination status within individual SARS-CoV-2 lineages (BA.1, BA.2, BA.5.1, XBB.1.5, XBB.1.9.1, and XBB.1.9.2), χ² test was applied. The category “missing” was excluded from the analysis when a variable had less than 5% missing values. For variables with more than 20% missing values, the analysis was conducted both with and without these values. In order to compare disease severity between SARS-CoV-2 variants hospitalization was used as an outcome and regression analyses were performed. To this end, a data subset excluding COVID-19 cases with missing values in any of the covariates was generated (Fig. 1). Independent variables included sex assigned at birth, age group, SARS-CoV-2 lineages, and the month of sampling date of the diagnostic sample. Univariate logistic regression models were fitted to examine the associations of each independent variable and the dichotomous outcome (hospitalization or non-hospitalization), and unadjusted odds ratios (OR) are presented. To account for potential confounding, multivariable logistic regression was used to analyze the association between multiple independent variables and the dichotomous outcome, with adjusted ORs (adjOR) presented. To assess the robustness of results, a sensitivity analysis was performed, including all sublineages of BA.1, BA.2, BA.5, BQ.1, XBB.1.5, XBB.1.9.1, and XBB.1.9.2 in the analyses on infection distribution and odds of hospitalization.

The non-parametric, two-tailed Spearman correlation test was used to analyze statistical correlation of SARS-CoV-2 lineage distribution captured by the IMSSC2 laboratory network and the DESH platform (*p < 0.05; **p < 0.01; ***p < 0.001; *p* < 0.0001). Statistical analyses of experimental data were performed using a non-paired, non-parametric Kruskal-Wallis test (*p < 0.05; **p < 0.01; ***p < 0.001) in GraphPad Prism Software Version 9.1.0. Results were presented as mean ± standard error (SEM).

### Ethical declaration

All investigations were carried out in accordance with the principles set forth in the Helsinki Declaration. Only pseudo-anonymized surveillance data were analyzed. For the analysis of surveillance data from the mandatory notification system, an ethical statement is not required according to the German Infection Protection Act. The linkage and processing procedures of epidemiological, clinical, and genomic data were conducted in compliance with §13(3) of the German Infection Protection Act, which permits the transfer of pathogen material and associated pseudonymized case data to designated institutions such as RKI, for surveillance and further epidemiological analyses. Epidemiological analyses were conducted in compliance with the STrengthening the Reporting of OBservational studies in Epidemiology (STROBE) guidelines.

## Results

### SARS-CoV-2 sublineage analysis

**Fig. 2:**
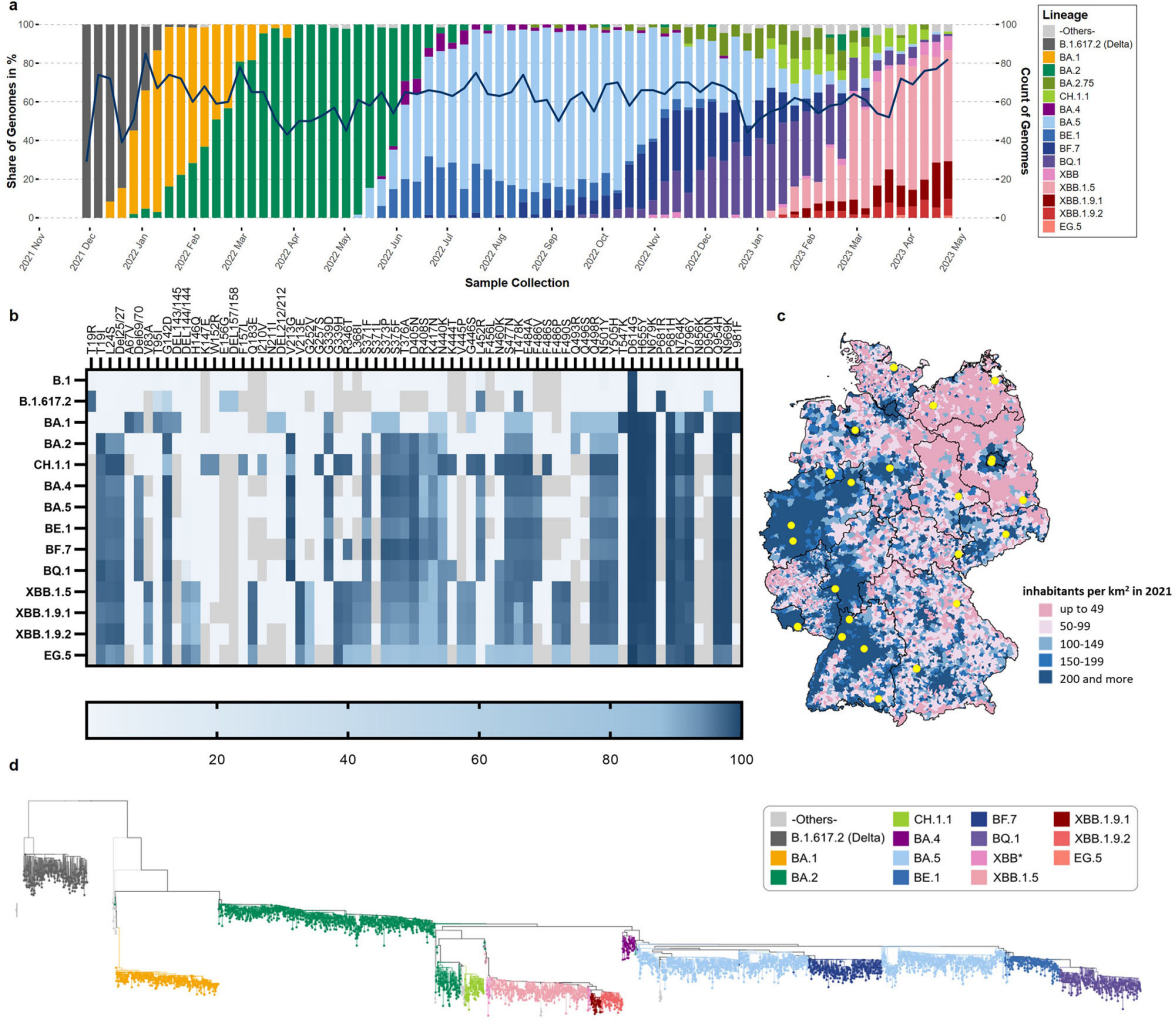
Distribution and Phylogenetic Analysis of SARS-CoV-2 Sublineages in Germany, December 1st, 2021, to April 30th, 2023, identified by the IMSSC2 laboratory network. **a** Stacked bar chart showing the chronological distribution of lineages for IMSSC2 samples (*n* = 4595). Colors represent the Delta (B.1.617.2) variant, including all its sublineages, and Omicron sublineages, including recombinants that emerged over the observation period. The solid line indicates the total count of samples sequenced within the IMSSC2 laboratory network on a weekly basis. The stacked bar plot was created in R (version 4.2.2) using the ggplot2 library (version 3.4.0). **b** Mutational profiles of SARS-CoV-2 sublineages illustrating mutation patterns in the spike protein of the respective Omicron sublineages, based on a minimal mutation prevalence of 75%, according to outbreak.info.org. **c** Map of Germany representing the geographic distribution of IMSSC2 network laboratories (highlighted in yellow) according to population density. The map is adapted from https://www.deutschlandatlas.bund.de/DE/Karten/Wo-wir-leben/006-Bevoelkerungsdichte.html**d** Phylogenetic analysis of German SARS-CoV-2 genomes identified by the IMSSC2 laboratory network. The phylogenetic tree, colored according to Pangolin lineage assignment and with branch length representing genetic divergence, is based on sequences randomly collected (*n* = 4,595).

During the time period covered by this study, from December 1st, 2021, to April 30th, 2023, a total of 4663 randomly selected samples were sent to the RKI for processing and WGS, of which 4,595 (98.54%) full-genomes were obtained. Samples originated from 24 network laboratories dispersed across Germany in 14 federal states. The distribution and number of laboratories reflects the population density as well as the number of COVID-19 cases as reported by the German mandatory notification system of the respective states (Fig. 2c, Supplementary Fig. 1). Within the study period, the original Omicron lineage (B.1.1.529) diversified into a wide range of phylogenetically related sublineages with diverse mutational profiles (Fig. 2b, d, Supplementary Fig. 2).

Figure 1a displays the dynamic changes in lineage compositions over time. Delta, the predominant variant in December 2021, was assigned to 270 (5.9%) genomes, whereas 4325 (94.1%) were classified as Omicron. Most prevalent Delta sublineages sequenced and identified with at least 5% frequency among all Delta sequences belonged to Pangolin lineages B.1.617.2 (8.1%), AY.4 (13.0%), AY.43 (30.4%), AY.121 (5.9%), AY.122 (16.7%), and AY.126 (5.6%). In January 2022, the Delta variant was replaced by Omicron sublineages BA.1 and BA.2, which prevailed through May 2022. In April and early May 2022, BA.2 accounted for > 95% of genome sequences. Subsequently, the Omicron BA.5 variant, including the descendant sublineages BA.5.1, BE.1, BF.7, and BQ.1, predominated through early February 2023, accounting for > 95% of genomes from mid-July to mid-November 2022. BA.2.75 and its sublineage CH.1.1 circulated in parallel with BA.5 and XBB sublineages with a combined prevalence of 8.3 – 27.4% between January and March 2023. XBB, a recombinant of the BA.2 progenies BJ.1 (BA.2.10.1) and BA.2.75^28,29^, emerged in January 2023. XBB sublineages, including XBB.1.5, XBB.1.9.1, and XBB.1.9.2, increased in prevalence and became predominant ( > 65%) in March 2023.

**Fig. 3:**
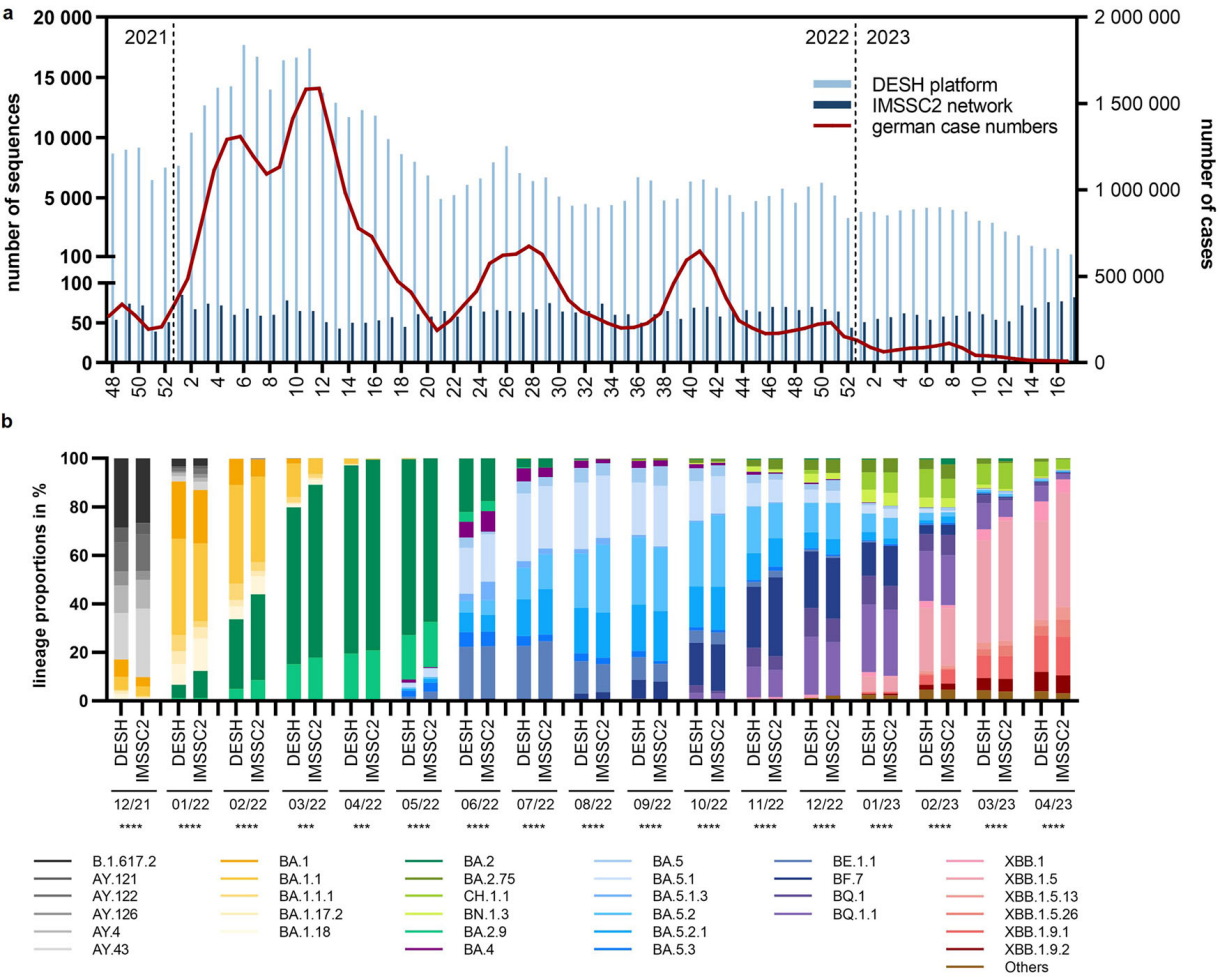
Comparison of SARS-CoV-2 lineage distribution captured by the IMSSC2 laboratory network and the DESH Platform. **a** The number of sequenced genomes captured by the IMSSC2 laboratory network and through the DESH platform from December 1st, 2021, to April 30th, 2023. The solid line represents reported case numbers in Germany according to the German Infection Protection Act. **b** Comparison of the SARS-CoV-2 sublineage proportions with at least 5% share identified in one of the indicated months, either in the IMSSC2 dataset or in the DESH dataset, which is two orders of magnitude larger, during the observation period from December 2021 to April 2023. Both datasets were statistically analyzed using non-parametric, two-tailed Spearman correlation test, with ***p < 0.001; ****p < 0.0001. Spearman correlation coefficients ρ in the individual months were between 0.62 and 0.99 indicating strong correlation of the IMSSC2 and DESH dataset. The analyses include only genome sequences that were obtained from randomly sampled specimens (DESH dataset: 511,533 sequences, IMSSC2 dataset: 4595 sequences).

Over the course of this study, Germany operated two complementing nationwide SARS-CoV-2 genomic surveillance instruments: (i) the IMSSC2 laboratory network and (ii) the DESH platform as part of the pandemic response^9^. We investigated whether the sequence diversity captured by the IMSSC2 laboratory network was representative in comparison to the more than 100-fold larger DESH dataset from the same time period (Fig. 3a). To this end, we determined the lineage proportions in the IMSSC2 dataset and compared them to those in the DESH dataset from the corresponding time period. For each of the 17 months assessed, we found a significant strong correlation (Spearman correlation coefficient ρ = 0.62–0.99; *p* ≤ 0.0002; Fig. 3b) between both datasets regarding the overall lineage distribution and the hierarchical ranking of predominant lineages. Additionally, we observed a comparable temporal course, particularly evident in the first detection of a sublineage and its acquisition of predominance within the same month (Fig. 3b).

### COVID-19 cases infected with prevalent SARS-CoV-2 variants

To gain further insight into the distribution of various SARS-CoV-2 lineages within the population, we analyzed epidemiological data from COVID-19 cases provided by the German mandatory notification system that were linked to SARS-CoV-2 genome sequences obtained either through the IMSSC2 laboratory network or submitted to the DESH platform.

During the study period, 272,770 (0.8%) of all notified COVID-19 cases (*n* = 32,468,122) were linked to genome data (Fig. 1 and Table S1). Of these, 84,639 (31.0%) cases infected with selected SARS-CoV-2 lineages — BA.1, BA.2, BA.5.1, BQ.1.1, XBB.1.5, XBB.1.9.1, and XBB.1.9.2 — were included in the analysis.

**Fig. 4:**
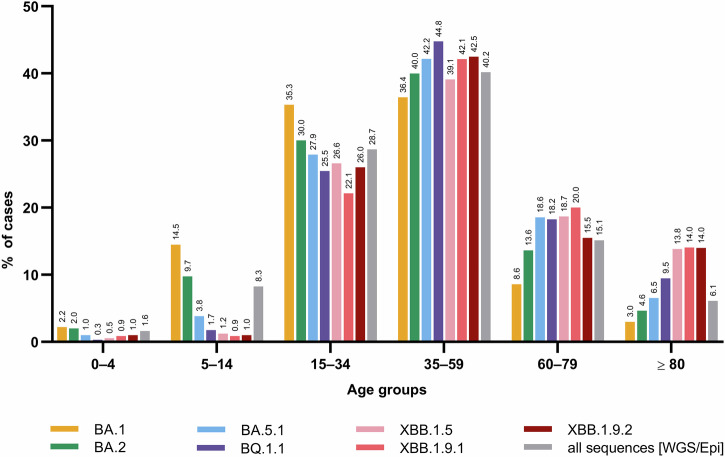
Distribution of COVID-19 cases among age groups. Relative frequency (%) of laboratory-confirmed BA.1, BA.2, BA.5.1, BQ.1.1, XBB.1.5, XBB.1.9.1, and XBB.1.9.2 cases among age groups in Germany between December 1st, 2021 and April 30th, 2023. The grey bars (all sequences [WGS/Epi]) within the age groups refer to all notified COVID-19 cases with epidemiological information and linked to full-genomes (*n* = 272,770).

Demographic and clinical characteristics of cases by lineage are summarized in Table 1. Most infections were attributed to the Omicron lineages BA.2 (67.0%) and BA.5.1 (15.4%). Overall, the dataset contains a significantly higher proportion of females than males (44,763 [52.9%] vs. 39,638 [46.8%], *p* < 0.001). In particular, significantly more females than males were reported to be infected with lineages BA.2 (*p* < 0.001), BA.5.1 (*p* = <0.001), BQ.1.1 (*p* < 0.001), and XBB.1.5 (*p* < 0.001), whereas no significant sex differences were found for those infected with BA.1 (*p* = 0.18), XBB.1.9.1 (*p* = 0.65) and XBB.1.9.2 (*p* = 0.94). Regarding age distribution, the proportion of cases among younger individuals (0–4 and 5–14 years) was higher for BA.1 and BA.2 compared to the other SARS-CoV-2 lineages within the same age groups (Table 1 and Fig. 4) – a pattern consistent with the results based on the IMSSC2 laboratory network only (Supplementary Fig. 3). Data on the vaccination status was largely incomplete, with missing information in 75.1% (61.4–87.7%) of cases, and was therefore excluded from all further analyses (Table 1). During the study period, 261 individuals infected with the analyzed lineages died, 95.8% of whom were aged 60 years and older. The highest case fatality rate was observed for individuals infected with XBB.1.5 (1.0%) (Table 1). A greater number of hospitalizations was particularly observed following an infection with recombinant variants XBB.1.5, XBB.1.9.1, and XBB.1.9.2 compared to Omicron BA.1 and BA.2 variants, whereas the highest number of intensive care admissions occurred among BA.1 cases (Table 1).

**Table 1:** Characteristics of COVID-19 cases grouped by SARS-CoV-2 lineages BA.1, BA.2, BA.5.1, BQ.1.1, XBB.1.5, XBB.1.9.1, and XBB.1.9.2, Germany, December 1st, 2021, to April 30th, 2023 (*n* = 84,639). Cases for which epidemiological data were not available are categorized as missing and are presented in italics

Characteristic	BA.1		BA.2		BA.5.1		BQ.1.1		XBB.1.5		XBB.1.9.1		XBB.1.9.2		Total	
	Number	%	Number	%	Number	%	Number	%	Number	%	Number	%	Number	%	Number	%
**Total**	6825	8.1	56728	67.0	13058	15.4	5394	6.4	2199	2.6	235	0.3	200	0.2	84639	100
**Sex**^**a**^																
Female	3453	50.6	30146	53.1	6832	52.3	2911	54.0	1201	54.6	121	51.5	99	49.5	44763	52.9
Male	3343	49.0	26427	46.6	6188	47.4	2469	45.8	997	45.3	114	48.5	100	50.0	39638	46.8
*Missing*	*29*	*0.4*	*155*	*0.3*	*38*	*0.3*	*14*	*0.3*	*1*	*0.05*	*0*	*0.0*	*1*	*0.5*	*238*	*0.3*
**Age groups (years)**																
0–4	151	2.2	1129	2.0	131	1.0	17	0.3	12	0.5	2	0.9	2	1.0	1444	1.7
5–14	989	14.5	5524	9.7	501	3.8	94	1.7	27	1.2	2	0.9	2	1.0	7139	8.4
15–34	2411	35.3	17019	30.0	3641	27.9	1373	25.5	585	26.6	52	22.1	52	26.0	25133	29.7
35–59	2486	36.4	22675	40.0	5508	42.2	2414	44.8	860	39.1	99	42.1	85	42.5	34127	40.3
60–79	585	8.6	7733	13.6	2423	18.6	984	18.2	411	18.7	47	20.0	31	15.5	12214	14.4
≥ 80	203	3.0	2633	4.6	854	6.6	511	9.5	304	13.8	33	14.0	28	14.0	4566	5.4
*Missing*	*0*	*0*	*15*	*0.03*	*0*	*0*	*1*	*0.02*	*0*	*0*	*0*	*0.0*	*0*	*0*	*16*	*0.02*
**Hospitalization**																
Yes	99	1.5	818	1.4	398	3.0	371	6.9	255	11.6	26	11.1	28	14.0	1995	2.4
No	3099	45.4	21494	37.9	4698	36.0	1682	31.2	632	28.7	58	24.7	60	30.0	31723	37.5
*Missing*	*3627*	*53.1*	*34416*	*60.7*	*7962*	*61.0*	*3341*	*61.9*	*1312*	*59.7*	*151*	*64.3*	*112*	*56.0*	*50921*	*60.2*
Intensive Care Unit*	n/N	%	n/N	%	n/N	%	n/N	%	n/N	%	n/N	%	n/N	%	n/N	%
Yes	13/99	13.1	75/818	9.2	32/398	8.0	22/371	5.9	10/255	3.9	2/26	7.7	1/28	3.6	155/1995	7.8
No	74/99	74.7	688/818	84.1	350/398	87.9	340/371	91.6	240/255	94.1	24/26	92.3	26/28	92.9	1742/1995	87.3
*Missing*	*12/99*	*12.1*	*55/818*	*6.7*	*16/398*	*4.0*	*9/371*	*2.4*	*5/255*	*2.0*	*0/26*	*0*	*1/28*	*3.6*	*98/*1995	*4.9*
**Deaths**																
Yes	20	0.3	138	0.2	43	0.3	36	0.7	23	1.0	1	0.4	0	0	261	0.3
No	6496	95.2	53801	94.8	12435	95.2	5208	96.6	2110	96.0	229	97.4	194	97.0	80473	95.1
*Missing*	*309*	*4.5*	*2789*	*4.9*	*580*	*4.4*	*150*	*2.8*	*66*	*3.0*	*5*	*2.1*	*6*	*3.0*	*3905*	*4.6*
**Vaccination**																
Yes	1997	29.3	11896	21.0	2538	19.4	979	18.1	313	14.2	27	11.5	25	12.5	17775	21.0
No	638	9.3	2323	4.1	234	1.8	65	1.2	35	1.6	2	0.9	5	2.5	3302	3.9
*Missing*	*4190*	*61.4*	*42509*	*74.9*	*10286*	*78.8*	*4350*	*80.6*	*1851*	*84.2*	*206*	*87.7*	*170*	*85.0*	*63562*	*75.1*

^a^Sex at birth. Due to the low sample size, cases with sex recorded as ‘diverse’ (n = 8) were considered missing and were not subject to further analysis.

^*^n = COVID-19 cases in intensive care unit, N = number of hospitalized COVID-19 cases.

A multivariable logistic regression model was employed, adjusting for sex, age group, and SARS-CoV-2 lineages (BA.1, BA.2, BA.5.1, BQ.1.1, XBB.1.5, XBB.1.9.1, and XBB.1.9.2) to estimate the odds for hospitalization and assess the effect of aforementioned factors. The month of diagnostic sampling was included to adjust for temporal variations (e.g., changes in testing and/or reporting). Characteristics of COVID-19 cases included in the subanalysis on hospitalization are summarized in Table S3. The hospitalization rates among patients in the subanalysis and the entire dataset were comparable (Tables S3 and, 1). Both unadjusted and adjusted analyses identified male sex and increasing age as statistically significant predictors of hospitalization (Table 2). Stratification by age groups revealed the highest odds of hospitalization in COVID-19 cases aged 0-4 years and 60 and above. In contrast, 5–59-year-olds had the lowest odds. Unadjusted odds ratios indicated that all lineages, particularly XBB.1.5, XBB.1.9.1, and XBB.1.9.2 were strongly associated with hospitalization compared to BA.2. This association was also observed in an adjusted model in which only sex and age were included (XBB.1.5: OR 6.91 [6.11–7.81], XBB.1.9.1: OR 7.42 [5.62––9.77] and XBB.1.9.2: OR 6.84 [4.80–9.70], data not shown). Notably, after further adjusting for the factor “month of diagnostic sampling” in the multivariable model, the association between most SARS-CoV-2 lineages and hospitalization observed in the unadjusted regression model was lost (Table 2). Compared to BA.2, BA.1 remained the only lineage significantly associated with hospitalization. In contrast, the lineages BQ.1 and XBB.1.5 were associated with significantly decreased odds of hospitalization after adjustment. These findings suggest that temporal factors, especially towards the end of our study period, may have influenced the association of lineages and hospitalization in the unadjusted regression model. This influence also persisted in an expanded dataset, which includes all sublineages of BA.1, BA.2, BA.5, BQ.1, XBB.1.5, XBB.1.9.1, and XBB.1.9.2 (data not shown).

**Table 2:** Logistic regression analyses of the association of hospitalization and sex, age group, SARS-CoV-2 lineages, and month of diagnostic sampling among COVID-19 cases, December 1st, 2021, to April 30th, 2023 (*n* = 33,632)

Categories	n/N^#^	%	Unadjusted OR (95% CI)	Adjusted OR (95% CI)
**Sex**				
Female	963/17935	5.4	**Reference**	**Reference**
Male	1032/15697	6.6	1.24 (1.13–1.36)***	1.50 (1.35–1.67)***
**Age group (years)**				
0–4	24/538	4.5	**Reference**	**Reference**
5–14	21/2739	0.8	0.17 (0.09–0.30)***	0.18 (0.10–0.33)***
15–34	129/9840	1.3	0.28 (0.19–0.45)***	0.25 (0.16–0.40)***
35–59	259/13149	2.0	0.43 (0.29–0.68)***	0.36 (0.24–0.57)***
60–79	648/5054	12.8	3.15 (2.12–4.91)***	2.43 (1.62–3.83)***
≥80	914/2312	39.5	14.0 (9.43–21.83)***	10.42 (6.94–16.39)***
**SARS-CoV-2 lineage**				
BA.1	99/3188	3.1	0.84 (0.68–1.03)	1.42 (1.02–1.96)*
BA.2	818/22258	3.7	**Reference**	**Reference**
BA.5.1	398/5085	7.8	2.23 (1.96–2.52)***	0.70 (0.46–1.08)
BQ.1.1	371/2042	18.2	5.82 (5.09–6.64)***	0.56 (0.33–0.96)*
XBB.1.5	255/887	28.7	10.58 (8.99–12.41)***	0.42 (0.23–0.78)**
XBB.1.9.1	26/84	31.0	11.75 (7.25–18.55)***	0.53 (0.23–1.23)
XBB.1.9.2	28/88	31.8	12.23 (7.66–19.07)***	0.45 (0.20–1.02)
**Month of diagnostic sampling**				
2021-12	11/491	2.2	**Reference**	**Reference**
2022-01	48/2268	2.1	0.94 (0.51–1.93)	0.89 (0.46–1.86)
2022-02	156/3901	4.0	1.82 (1.03–3.58)	1.38 (0.72–2.89)
2022-03	298/7863	3.8	1.72 (0.98–3.36)	1.36 (0.70–2.89)
2022-04	227/6187	3.7	1.66 (0.95–3.26)	1.18 (0.60–2.52)
2022-05	134/3904	3.4	1.55 (0.87–3.06)	1.18 (0.59–2.53)
2022-06	60/1770	3.4	1.53 (0.83–3.1)	1.30 (0.61–2.92)
2022-07	66/1431	4.6	2.11 (1.15–4.25)*	2.13 (0.93–5.12)
2022-08	93/940	9.9	4.79 (2.65–9.56)***	4.08 (1.78–9.88)**
2022-09	71/838	8.5	4.04 (2.21–8.13)***	3.96 (1.71–9.68)**
2022-10	89/625	14.2	7.25 (4.00–14.51)***	6.82 (2.96–16.61)***
2022-11	76/625	12.2	6.04 (3.31–12.15)***	6.25 (2.66–15.48)***
2022-12	126/807	15.6	8.07 (4.51–16.02)***	7.99 (3.36–19.95)***
2023-01	129/649	19.9	10.83 (6.05–21.49)***	13.48 (5.75–35.00)***
2023-02	165/713	23.1	13.14 (7.39–25.97)***	15.52 (6.40–39.53)***
2023-03	172/485	35.5	23.98 (13.43–47.53)***	26.35 (10.48–69.41)***
2023-04	74/135	54.8	52.94 (27.67 – 110.59)***	42.88 (15.84 – 120.98)***

# *n* number of hospitalizations, *N* number of infected cases reported to the German notification system.

*CI* confidence interval, *OR* odds ratio.

*p value: p < 0.05: *p < 0.01: **p < 0.001: ***

Univariate models were fitted for each independent variable separately, with unadjusted odds ratios (ORs) presented. Multivariable model included all independent variables simultaneously and provide adjusted ORs.

### Rapid propagation of predominating SARS-CoV-2 Omicron sublineages in polarized human primary cell cultures grown at the ALI of the URT

To complement the epidemiological findings with viral phenotypes and to evaluate their epidemic potential, we investigated representative SARS-CoV-2 isolates for immunoevasive properties and for replication in human infection models. Therefore, we cultured SARS-CoV-2 lineages BA.2, BA.5.1, BQ.1.1, and recombinant sublineages XBB.1.5, XBB.1.9.1, and XBB.1.9.2 from patient samples. For comparison, we used an early SARS-CoV-2 isolate (D614G) and a representative Delta variant (B.1.617.2) that predominated at the beginning of the study period.

The ongoing evolution of SARS-CoV-2 is particularly driven by improved transmissibility and/or the acquisition of immunoevasive properties. In order to evaluate the latter aspect, we measured the neutralization efficiency of a pre-Omicron WHO serum pool against each of the isolated SARS-CoV-2 variants. Relative to the D614G virus, neutralization titers were decreased for all variants. While the Delta Variant of Concern (VOC), BA.2, and BA.5.1 featured a four to seven-fold reduction in titer, titer reductions were much more pronounced for Omicron sublineages which had emerged subsequently, i.e., BQ.1.1 (84-fold), XBB.1.5 (134-fold), and XBB.1.9.2 (224-fold) (Supplementary Fig. 4).

To get first impressions of potential changes in the growth behavior of newly emerged viruses, the respective virus isolates were compared on different culture systems. Vero E6 cells are a commonly used infection model for coronaviruses and were employed for a first comparative analysis of virus variants. The Delta variant and also all Omicron sublineages showed delayed replication kinetics compared to the D614G, with viral titers below those of D614G by one to almost three orders of magnitude at 16 h p.i. (Supplementary Fig. 5). Area under the curve analyses (AUC) indicated decreased replication, especially for SARS-CoV-2 sublineages BQ.1.1, XBB.1.5, XBB.1.9.1, and XBB.1.9.2 on Vero E6 cells (Supplementary Fig. 5d). Given that Vero E6 cells are a non-human primate kidney-derived cell line lacking expression of type I Interferon (IFN) genes, more physiologically relevant models representing different segments of the human respiratory tract were used for further analyses.

**Fig. 5:**
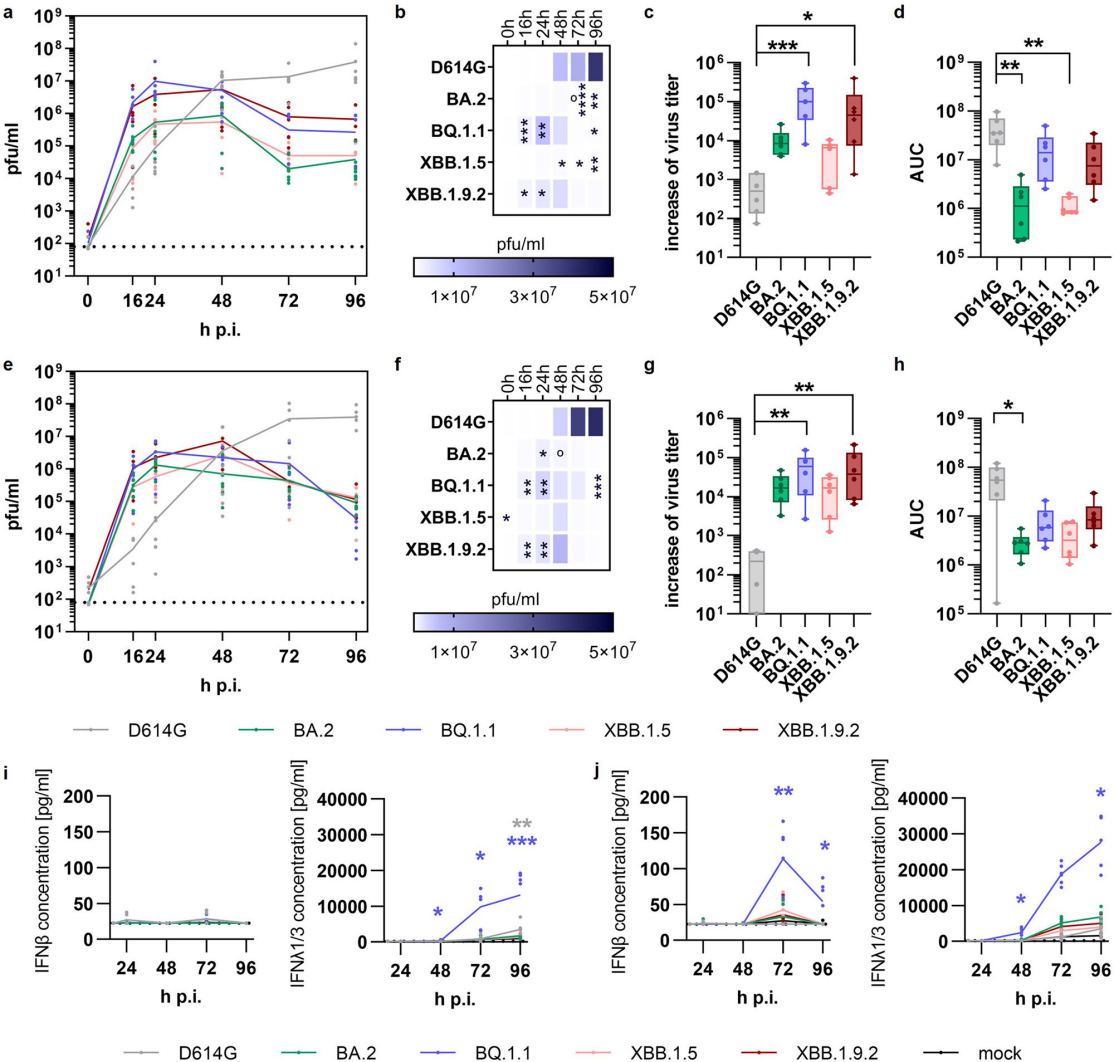
Phenotypic Characterization of Omicron Sublineages in Complex Models of the conducting airways. Primary human nasal (a-d, i, k) and bronchial (e-h, j-k) ALI cultures were infected with SARS-CoV-2 D614G and selected SARS-CoV-2 sublineages (BA.2, BQ.1.1, XBB.1.5, or XBB.1.9.2) at MOI 0.1. Experiments were conducted for three donors in technical duplicates. **a**, **e** Progeny viruses were collected by washes from the apical compartment at indicated time points and titrated using standard plaque assay on Vero E6 cells. Replication analyses are presented as mean ± SEM. **b**, **f** Heatmap represents viral titers from replication analyses (**a**) or (**e**) at corresponding time points. **c**, **g** Increase of viral titers during the early infection phase was calculated using linear regression between the two initial data points (0 and 16 h p.i.) from replication analyses (**a**) or (**e**). Data are shown as boxplots (min to max; box extends from the 25th to the 75th percentile, center line represents median). **d**, **h** Area under the curves (AUCs) were calculated from replication analyses shown in panels a and e, respectively, with data represented as mean ± SEM. For determination of immune activation, basolateral fluids were collected from nasal (**i**) or bronchial (**j**) ALI cultures at indicated time points and used for detection of type I and III IFN via ELISA. The limit of detection is marked by the dotted line. **a**–**f** Statistical analyses were performed using non-paired, non-parametric Kruskal-Wallis test, with *p < 0.05; **p < 0.01; ***p < 0.001. Statistical significance in (**b**) and (**f**) is displayed in comparison to SARS-CoV-2 D614G (*) or XBB.1.9.2 (O), and in (**i**) and (**j**) relative to mock infection.

Replication efficiency within the human conducting airways — one important factor underlying intrinsic transmissibility — was assessed with selected virus variants. To this end, we performed assays on reconstituted primary human nasal and bronchial epithelia, which were infected with SARS-CoV-2 variants BA.2, BQ.1.1, XBB.1.5, XBB.1.9.2, or the early D614G isolate. This replication analysis revealed an enhanced growth phenotype of Omicron sublineages at early time points in both nasal and bronchial ALI cultures (Fig. 5). That effect was significantly pronounced for BQ.1.1 and XBB.1.9.2, exceeding viral titers of the D614G virus by more than two orders of magnitude at 16 h p.i. (Fig. 5a, b (nasal) and e, f (bronchial)). Calculation of the linear increase of viral replication during the early infection phase (0 – 16 h p.i.) confirmed this early growth advantage with up to 326-times steeper increases for Omicron sublineages (Fig. 5c, g). Interestingly, although propagation of the D614G virus displayed a pronounced delay compared to Omicron sublineages, its peak titers eventually surpassed those of Omicron sublineages BQ.1.1 and XBB.1.9.2 up to 1330-fold at 96 h p.i.. As a result, the AUC of the early D614G isolate exceeded that of the SARS-CoV-2 sublineages, despite their initial enhanced increase in viral titers (Fig. 5d, h).

To gain insight into the innate immune responses induced by the different sublineages, we measured type I and III IFN responses in infected primary URT cultures. While little to no IFN release was observed at 16 h and 24 h p.i., Omicron sublineage BQ.1.1 induced a significantly increased secretion of type-I and type-III-IFN at 72 h and 96 h p.i. (Fig. 5i, j). Even though BQ.1.1 and XBB.1.9.2 viruses showed similar replication kinetics, BQ.1.1 induced higher IFN-λ1/3 levels compared to XBB.1.9.2 (Fig. 5i, j).

### Increased replication capacity of XBB.1.9.2 variant compared to former Omicron sublineages in human lung cell cultures

**Fig. 6:**
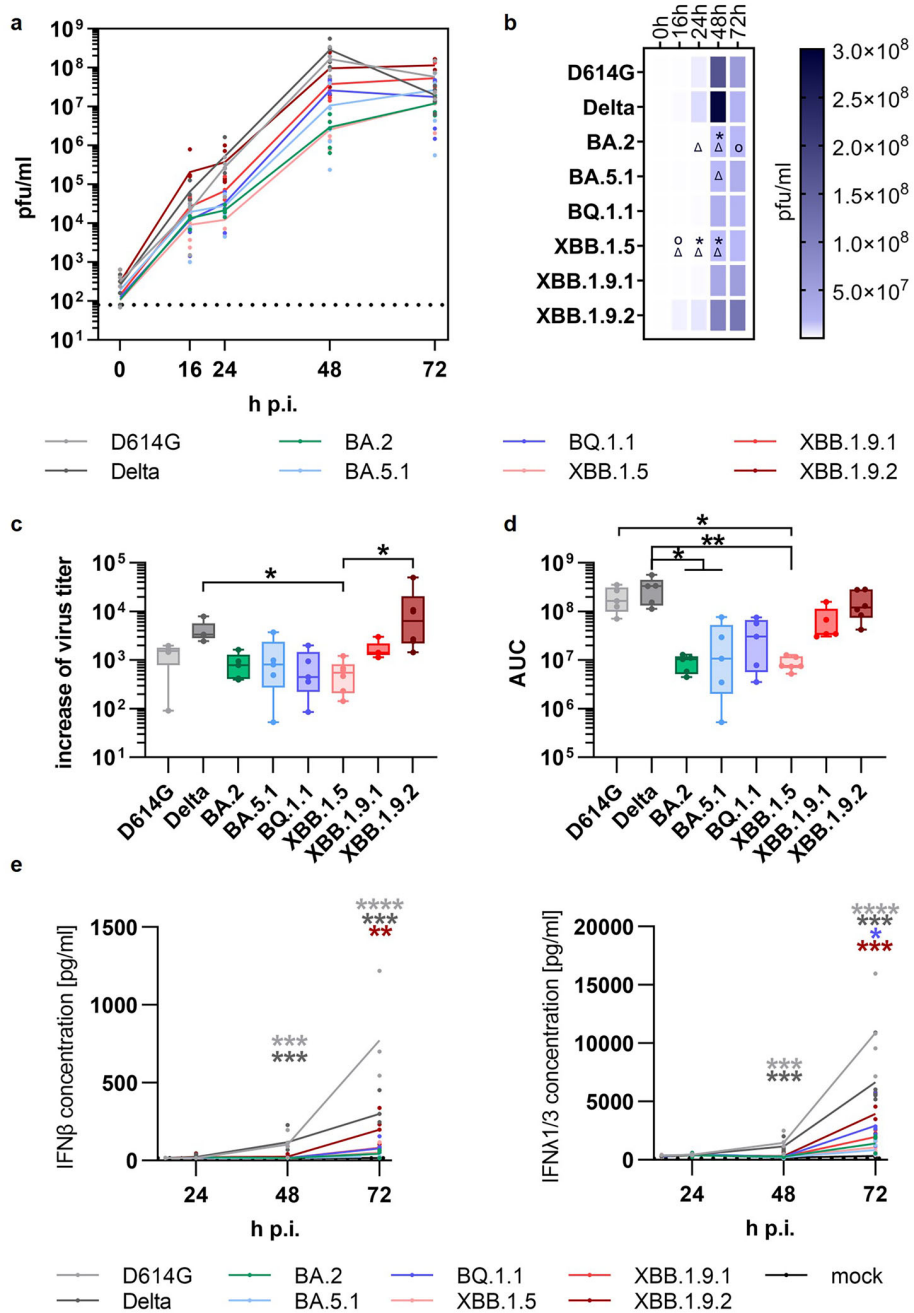
Phenotypic Characterization of Omicron Sublineages in a Human Alveolar Infection Model. hAELVi cells grown under Air-Liquid Interface (ALI) conditions for 21 days were infected with the indicated SARS-CoV-2 viruses: D614G, Delta (B.1.617.2), and selected Omicron sublineages (BA.2, BA.5.1, BQ.1.1, XBB.1.5, XBB.1.9.1, or XBB.1.9.2) at MOI 0.1. Analyses were performed in three independent experiments in technical duplicates. **a** Progeny viruses were collected by washes from the apical compartment at the indicated time points and titrated using standard plaque assay on Vero E6 cells. Replication analysis is shown as mean ± SEM. **b** Heatmap representing viral titers from replication analysis (a) at corresponding time points. **c** The increase in viral titers during the early phase of infection was calculated from linear regression between the two initial data points (0 and 16 h p.i.) from replication analysis shown in (**a**). Data are presented as boxplots (min to max; box extends from the 25th to the 75th percentile, center line represents median). **d** Area under the curves (AUCs) were calculated from the replication analysis in (a). Data are shown as mean ± SEM. **e** For determination of immune activation, basolateral fluids were collected at indicated time points and analyzed for the detection of type I and III IFN via ELISA. The limit of detection is indicated by the dotted line. **a**–**e** Statistical analyses were performed using a non-paired, non-parametric Kruskal-Wallis test, *p < 0.05; **p < 0.01; ***p < 0.001. Statistical significances in (**b**) are displayed in comparison to SARS-CoV-2 D614G (*), Delta (Δ), or XBB.1.9.2 (O), and in (**e**) compared to mock infection.

Polarized hAELVi lung cell cultures were infected with selected SARS-CoV-2 Omicron sublineages to evaluate relative viral fitness in cells of the distal lung. Interestingly, the XBB.1.9.2 variant demonstrated 3- to 10-fold higher titers compared to the D614G virus and the Delta variant, and up to two orders of magnitude higher than earlier Omicron sublineages in the early course of infection at 16 h p.i. (Fig. 6a, b). This finding was further supported by the steepest increase of viral replication calculated between 0 h and 16 h p.i. for XBB.1.9.2 which exceeded that of the D614G virus by 11.5-fold (Fig. 6c). Additionally, AUC calculations indicated a significantly reduced replication capacity for SARS-CoV-2 sublineages BA.2 and XBB.1.5, while least reduction was observed for the recombinant XBB.1.9.2 (Fig. 6d).

We further evaluated the variants for immune activation by determining IFN release into the basolateral compartment. We observed the most substantial IFNß- and IFNλ1/3 inductions for D614G and Delta, starting at 48 h p.i. In comparison, all Omicron sublineages showed relatively little IFN induction at all timepoints tested, with the strongest expression observed for the XBB.1.9.2 virus at 72 h p.i. (Fig. 6e).

## Discussion

The continuous emergence of novel, virulent SARS-CoV-2 variants characterized by increased transmissibility and pronounced immune evasion underscores the importance of monitoring these viral pathogens also in the post-pandemic era^10^. Here, we present the results of 17 months of SARS-CoV-2 integrated genomic surveillance in Germany, with genomic, virological, and epidemiological data collected from 4595 SARS-CoV-2 infections occurring between December 1st, 2021, and April 30th, 2023. This period includes the decline of the Delta wave, followed by the emergence of several Omicron sublineages, including BA.1, BA.2, BA.2.75, and various BA.5 descendants. The study period concludes with the predominance of recombinant XBB viruses in early 2023. The distribution and emergence of new SARS-CoV-2 variants observed in this dataset mirror the lineage proportions over time documented in other European and even North American countries^30–36^.

In 2020, the RKI initiated the IMSSC2 laboratory network to complement the mandatory national epidemiological surveillance with a representative and resource-efficient SARS-CoV-2 genomic surveillance instrument. This tool has been continuously operational since its inception. Here, we compare data from the IMSSC2 laboratory network to the more than 100-fold larger DESH dataset, which was obtained through Corona Surveillance Regulation as part of the ad-hoc pandemic response. The latter mechanism enabled the centralized, compensated collection of over one million SARS-CoV-2 genomes from diagnostic laboratories between January 2021 and April 2023 via the DESH portal^9^. Given the high costs associated with maintaining large-scale genomic surveillance, the long-term operation of DESH was never intended. However, the IMSSC2 laboratory network remains active following the conclusion of the large-scale remunerated genomic surveillance in April 2023. In this study, we investigated whether the sequence diversity captured by the IMSSC2 laboratory network was representative when compared to the DESH dataset. We observed a highly significant correlation in terms of the ranking of predominant lineages and lineage distribution between the IMSSC2 and DESH datasets during the period covered by this study. This correlation was also observed in an earlier study, where the abundance of major variants was captured quite accurately when compared to the GISAID dataset^12^. Our findings suggest that the centralized IMSSC2 laboratory network approach is well-suited to describe the phylodynamics of SARS-CoV-2 in a pandemic and post-pandemic period and can serve as a hub for systematic, lab-based downstream risk assessment.

To gain further insights into the spread of SARS-CoV-2 variants within the population, we analyzed epidemiological data reported by the mandatory national notification system for COVID-19 cases along with additional genomic information on SARS-CoV-2 lineages BA.1, BA.2, BA.5.1, BQ.1.1, XBB.1.5, XBB.1.9.1, and XBB.1.9.2. Despite the difference in absolute numbers, the smaller variant-specific dataset reflected the overall national case distribution obtained through the mandatory German national surveillance system, supporting its applicability for subsequent analyses. The majority of cases in our dataset were attributed to BA.2, which emerged at the beginning of the study period, while the fewest cases were associated with XBB.1.9.2 towards the end of this analysis.

With the emergence of Omicron, particularly early sublineages BA.1 and BA.2, studies on disease severity revealed higher incidences among children and young adults compared to pre-Omicron variants, such as Alpha or Delta^37^. These observations were hypothesized to be associated with multiple factors: approximately 3.2 times higher transmissibility of the Omicron variant compared to the Delta variant^38^, lower vaccination rates among children, as well as the termination of non-pharmaceutical interventions (NPIs, e.g., school closures) when Omicron emerged^39^. Although BA.1 and BA.2 incidences were not directly compared to pre-Omicron VOCs, a higher incidence of BA.1 and BA.2 infections was found in children (ages 0–14) than in other lineages within the same age groups in this study, which is consistent with findings from other studies^40,41^. Interestingly, for sublineages evolving after BA.1 and BA.2, infected individuals were more frequently from older age groups, which was also observed by others^40^. This trend continued for XBB recombinants that originated from two BA.2 sublineages. These observations might be explained by changes in population immunity, including increased immunity in children due to previous infection and/or vaccine availability for children in early 2022^42,43^. Indeed, by mid-2022, a large proportion of children and adolescents in Germany had acquired infection-induced or hybrid immunity following widespread BA.1/BA.2 circulation^44,45^. In contrast, waning immunity among vaccinated adults (78% with booster vaccination) may have contributed to the higher proportion of infections observed in older age groups. Also, the Omicron complex of lineages is antigenically distinct from the ancestral SARS-CoV-2 strain and previous VOCs, leading to an enhanced immune escape and to a reduced protection against infection^46–50^. Immune imprinting, whereby prior exposure to ancestral antigens shapes and potentially limits immune responses to antigenically distant variants, may also partly explain the shift towards older individuals during waves of later Omicron sublineages such as BA.5 and XBB^51–54^. However, the apparently higher proportions of XBB.1.9.1 infections in individuals aged 60–79 years in the IMSSC2 laboratory network data compared to the overall dataset is a consequence of the small sample size in this subgroup, resulting in proportionally inflated values.

Overall, significantly more females than males were observed to be infected. However, whether the higher proportion among females infected with lineages BA.2, BA.5.1, BQ.1.1 and XBB.1.5 reflects true biological differences in susceptibility or rather differences in healthcare-seeking behavior, testing frequency, or occupational exposure patterns remains unclear. Although males appeared to have been infected less frequently, uni- and multivariable logistic regression identified male sex as a significant predictor of hospitalization. This finding is consistent with previous studies that associated male sex with higher hospitalization rates across all age groups^55–57^. Another significant predictor in both logistic regressions was increasing age. In fact, age is a known risk factor for severe COVID-19 outcomes, particularly in individuals aged 60 and above^58^. In this study, hospitalization rates among children were higher in infants (0–4 years of age) compared to the 5–14-year-olds across all variants. However, the highest rates were observed among individuals over 60 years of age. The robustness of these findings was verified in a sensitivity analysis (data not shown).

The study on epidemiology data has its limitations. Since data on the cause of hospitalization (hospitalized “due to” versus “with” COVID-19) were not available, hospitalization rates in this study may be overestimated, particularly among children with an overall low number of reported cases and potential reporting biases. Additionally, the lack of data on comorbidities, which might affect hospitalization, limits interpretation. Importantly, no data on previous SARS-CoV-2 infections were available, and data on vaccination status were largely incomplete, which is a major limitation. Since both vaccination and infection history can substantially influence disease severity, considering vaccination status alone—without accounting for prior infections—could introduce bias. Consequently, the vaccination status was not included in the regression analysis on hospitalization. Thus, it remains unclear how previous infection or vaccination could have affected hospitalization and age distribution among COVID-19 cases. Changes in testing and reporting requirements during the study period also limit robust conclusions. In Germany, from March/April 2022 onward, regular testing frequency decreased substantially, which likely led to an underrepresentation of mild or asymptomatic infections in the mandatory surveillance data. The discontinuation of no-cost testing for all citizens at the end of 2022 may explain the significant decline of notified cases towards the end of the study period, leading to an underestimation of true overall case numbers and an overestimation of hospitalization^39,59^. As a consequence, later variants such as XBB.1.5, XBB.1.9.1, and XBB.1.9.2 might appear to have disproportionately high hospitalization rates. This interpretation is supported by the multivariable model, in which adjustment for calendar time (month of diagnostic sampling) attenuated lineage-specific differences in hospitalizations, with no increased odds of hospitalization for the recombinant XBB variants and even reduced odds for lineages BQ.1 and XBB.1.5. Thus, apparent lineage-specific differences in hospitalization are more likely reflecting reporting artefacts and temporal changes, e.g., lower outpatient consultation rates, behavioral changes, increased population immunity, rather than true virological differences in pathogenicity in a non-naïve population.

To be prepared for the emergence of new SARS-CoV-2 variants that may arise in the future and pose a significant risk to global public health, it is essential and recommended to continue risk assessment efforts as facilitated by continuous genomic analyses, as well as epidemiological and virological characterizations^60^. While genomic surveillance enables rapid identification of emerging lineages and epidemiological data captures their impact on the population, both approaches are not well able to distinguish intrinsic viral characteristics from effects driven by heterogeneous population immunity and/or population behavior as well as to evaluate the risk for vulnerable population groups with reduced or absent immunity. Therefore, a deeper understanding of phenotypes of SARS-CoV-2 variants, along with their intrinsic transmissibility and pathogenesis in the human respiratory tract, is required to support the interpretation of epidemiological observations. In particular, high infectious viral loads in the conducting airways may contribute to enhanced transmissibility due to increased shedding of infectious virus particles. Conversely, enhanced replication in the distal lung could indicate a greater capacity to induce severe disease. Reliable infection models that closely mimic viral replication and host cell activation in patients are highly desirable for studying viral infections of the human respiratory tract. While animal models are advantageous for studying complex pathophysiological questions due to the involvement of multiple cell types and an active immune system, their implementation and maintenance are both resource-intensive and time-consuming, especially under BSL3 conditions. Therefore, investigations in cell culture-based infection models of human respiratory origin may be a favorable choice for initial studies^61^.

In this study we utilized physiologically relevant experimental models representing different segments of the human respiratory tract, ranging from the conducting airways (including the nose and bronchi) to the distal lung to assess the virological properties of the SARS-CoV-2 variants predominant in 2022-23. We observed a faster replication phenotype but lower viral peak titers in the conducting airways for all Omicron sublineages investigated, compared to the early D614G virus (Fig. 5a–h), whereas most SARS-CoV-2 sublineages exhibited a reduced replication in differentiated human alveolar cells (Fig. 6). These observations confirm and extend those of other groups^14,36,62–72^.

However, we observed differences in replication capacities not only in comparison to the early D614G isolate, but also among different Omicron sublineages. In reconstituted nasal epithelia, BQ.1.1 and XBB.1.9.2 displayed slightly higher replication than BA.2 and XBB.1.5 (Fig. 5a–d). These data align with the more efficient replication of BA.5, the parental group of BQ.1.1, compared to BA.2 as reported by others^6,73^. Variations between Omicron sublineages are further supported by epidemiological data that report on remarkable differences in the clinical presentations among Omicron BA.1, BA.2, and BA.5 sublineages^40^.

Interestingly, we observed an increased replication phenotype for the XBB.1.9.2 variant in both conducting airway models and in the distal lung model. XBB.1.9.2 is the direct precursor of the EG.5 SARS-CoV-2 sublineage, a highly successful variant that additionally acquired the S456L mutation in the viral spike protein and became globally dominant in the second half of 2023. The replication phenotypes of XBB.1.9.2 observed in our study are comparable to data from Syrian hamsters infected with the XBB.1.9.2 descendant, EG.5^74^. Although speculative, an increased replication capacity in the lung might indicate that in an immunologically naïve population, this recombinant sublineage could cause increased pathology, similar to the precursor virus. This hypothesis is supported by the unadjusted odds ratios that suggested a strong association of XBB.1.9.2 with hospitalization compared to BA.2. However, this association was absent when integrating temporal factors, such as changes in testing and/or reporting of COVID-19 cases, and a changing population immunity over time into the model (Table 2).

It has been shown that, in addition to active viral replication, the induction of regulatory immune factors plays a critical role in the pathogenesis of COVID-19^75^. Investigation of innate immune activation showed the induction of type III IFN in human nasal and bronchial epithelial cultures with the most pronounced response observed for Omicron sublineages (Fig. 5i–k). This corresponds with data from a study that observed significant activation of IFNβ and IFNλ1 in primary nasal cultures^68^. A robust IFN response in the upper airways is associated with mild disease^75,76^. Therefore, the more pronounced induction of IFN following infection with Omicron variants may contribute to the milder disease typically associated with Omicron^77,78^.

Taken together, our in vitro phenotype assessment suggests ongoing adaptation of SARS-CoV-2 to the human respiratory tract, with the virus evolving for efficient replication in the conducting airways, which may be associated with increased intrinsic transmissibility^79^. This adaptation is particularly driven by the evolution of the viral spike protein, which has been observed across different SARS-CoV-2 variants and their sublineages throughout the pandemic. The F486V/P/S mutation emerged convergently in both Omicron BA.5- and XBB-sublineages. This mutation shifted the receptor-binding domain (RBD) conformation to a closed conformation, hiding the ACE2 binding site to evade neutralizing antibodies, which is supported by a less pronounced neutralization efficiency observed for Omicron sublineages in this study (Fig. S4) and by others^36,80–83^.

The COVID-19 pandemic has underscored the urgent need for robust and scalable surveillance systems that integrate genomic, epidemiological and virological data. Our results demonstrate that the IMSSC2 laboratory network, as a scalable, geographically representative surveillance infrastructure, is well-suited for early detection of emerging variants and efficient monitoring of SARS-CoV-2 evolution in the post-pandemic period. The collected genomic data reflect the diversification of Omicron into multiple sublineages. Importantly, our findings show that systematically collected, representative genomic data can capture viral evolution in high resolution, even with low numbers of samples. As such the network is a sustainable and cost-effective alternative to complement pandemic preparedness infrastructure. To assess the risk of emerging SARS-CoV-2 lineages comprehensively, it is advantageous to complement genomic and epidemiological data from the IMSSC2 laboratory network with continuous, laboratory-based, systematic evaluation of viral phenotypes, employing authentic viruses in physiologically relevant models. Thus, harmonized and sustainable reporting structures, interoperable data platforms, cross-sector collaboration, and rapid analysis capacities are essential to swiftly turn surveillance data into public health measures. Further, embedding integrated genomic surveillance into existing routine diagnostic settings will not only improve pandemic preparedness but also serve as a scalable model for monitoring of other respiratory pathogens.

## Supplementary information

Supplementary Information

## Data Availability

The program codes that were used to analyze epidemiological data exclusively use standard R packages and are available from the corresponding author upon request.

## Data availability

All Integrated Molecular Surveillance for SARS-CoV-2 (IMSSC2) genome sequences used in this study and underlying data to generate Figs. 2 to 6 are available on Zenodo^15^: All other datasets generated and analyzed during the current study are available from the corresponding authors on reasonable request.
