# Supplementary material for: Monitoring viral evolution and epidemiological characteristics of SARS-CoV-2 during 2022–2023 using Integrated Genomic Surveillance
Source: Commun Med (Lond). 2026 May 26;6:305. doi: 10.1038/s43856-026-01647-x (PMC13212648; doi:10.1038/s43856-026-01647-x)
Supplement: Supplementary file 1 — Supplementary Information [file 43856_2026_1647_MOESM1_ESM.docx]

**Supplementary Information**

**Monitoring Viral Evolution and Epidemiological Characteristics of SARS-CoV-2 during 2022-2023 using Integrated Genomic Surveillance**

**Authors**

Christin Mache^1,#^, Romy Kerber^2,#^, Jessica Schulze^1^, Marie Lataretu^3^, Susan Abunijela^2^, Yusra Seyam^3^, Sofia Paraskevopoulou^3^, Djin-Ye Oh^1^, Maximilian Arlt^1^, Aleksandar Radonić^3^, Somayyeh Sedaghatjoo^3^, Matthias Budt^1^, Ann-Sophie Lehfeld^2^, Felix Hartkopf^3^, Ralf Dürrwald^1^, Torsten Semmler^3^, Walter Haas^2^, Stephan Fuchs^3^, Stefan Kröger^2,+,*^, Thorsten Wolff^1,+,*^, Integrated Molecular Surveillance for SARS-CoV-2 (IMSSC2) Laboratory Network

^#^C.M. and R.K. contributed equally

^+^S.K. and T.W. contributed equally

**Affiliations**

^1^Influenza and other Respiratory Viruses (Unit 17), Robert Koch Institute, Berlin, Germany

^2^Respiratory Infections (Unit 36), Robert Koch Institute, Berlin, Germany

^3^Genome Competence Centre (MF1), Robert Koch Institute, Berlin, Germany

^*^Address for correspondence:

Thorsten Wolff, Robert Koch Institute, Seestr. 10, 13353 Berlin, Germany; email: [WolffT@rki.de](mailto:WolffT@rki.de)

Romy Kerber, Robert Koch Institute, Seestr. 10, 13353 Berlin, Germany; email: [KerberR@rki.de](mailto:KerberR@rki.de)


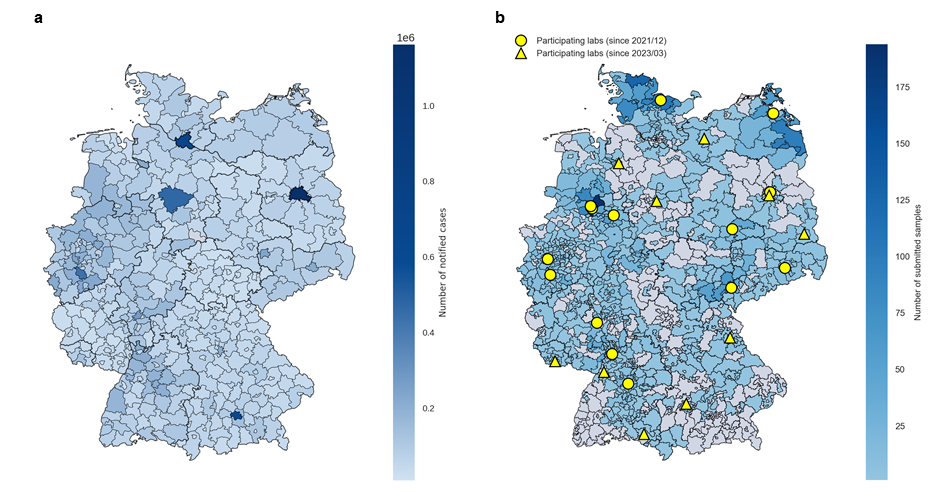


**Suppl. Figure 1. Geographical Distribution of SARS-CoV-2 Sequences Captured by the IMSSC2 Laboratory Network (12/2021 - 04/2023). a** Map of Germany showing the geographic distribution of SARS-CoV-2 cases notified to the mandatory German national surveillance system from December 1st, 2021, to April 30th, 2023 (n=32,468,122). Shades of blue indicate case numbers per county of notification. **b** Map of Germany showing the geographic distribution of SARS-CoV-2 positive samples included in the IMSSC2 genomic surveillance from December 1st, 2021, to April 30th, 2023 (n=4,595). Shades of blue indicate sample numbers per the 3-digit zip code region of origin, while IMSSC2 network laboratories are represented by yellow points (participating since 12/2021) or triangles (participating since 03/2023). The maps were created with a custom Python script using the GeoPandas package version 0.10.2^67^.

**
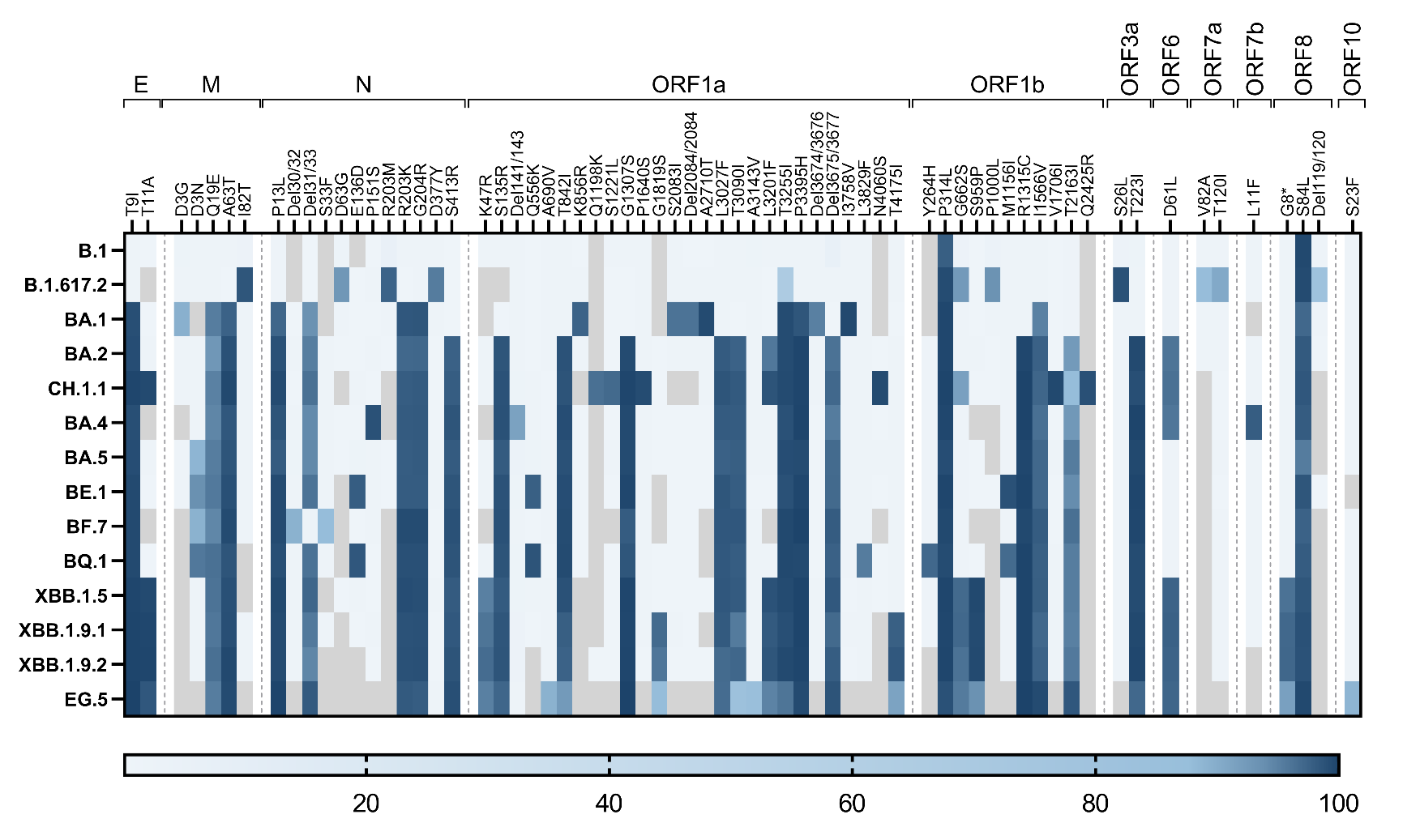
**

**Suppl. Figure 2. Mutational Profiles of Omicron Sublineages.** Mutation patterns of the indicated SARS-CoV-2 variants in the structural viral proteins E, M, and N, as well as the open reading frames (ORF) ORF1a, ORF1b, ORF3a, ORF6, ORF7a, ORF7b, ORF8, and ORF10, based on a minimum mutation prevalence of 75% according to outbreak.info.org.

**Suppl. Figure 3. Distribution of COVID-19 Cases among Age Groups in the IMSSC2 Laboratory Network.** Relative frequency (%) and total amount of laboratory confirmed BA.1, BA.2, BA.5.1, BQ.1.1, XBB.1.5, XBB.1.9.1, and XBB.1.9.2 cases among age groups calculated based on year of birth of COVID-19 case, in Germany between December 1st, 2021, to April 30th, 2023. The relative frequency of a SARS-CoV-2 lineage within an age group is based on the total number of cases detected for that lineage across all age groups through the IMSSC2 laboratory network (BA.1: n= 64, BA.2: n= 634, BA.5.1: n = 256, BQ.1.1: n = 126, XBB.1.5: n = 160, XBB.1.9.1: n = 37, XBB.1.9.2: n = 9).The grey bars (all sequences) within the age groups refer to all notified COVID-19 cases with epidemiological information and linked to full-genome sequences from the IMSSC2 laboratory network (n = 4,595).

**Suppl. Figure 4. Serum Neutralization Titers against Selected Omicron Sublineages.** Neutralization titers of pooled pre-Omicron sera (WHO reference serum panel NIBSC 21/338) against authentic SARS-CoV-2 viruses including D614G, Delta (B.1.617.2) and selected Omicron sublineages BA.2, BA.5.1, BQ.1.1, XBB.1.5 or XBB.1.9.2 are expressed as PRNT50 titers. PRNT50 of negative serum (NIBSC 20/142) was below detection limit in all assays (data not shown). Analyses were performed in three independent experiments in technical duplicates. Data are shown as geometric mean PRNT50 titer and 95% confidence intervals. Statistical analyses were performed using non-paired, non-parametric Kruskal-Wallis test (*p < 0.05; **p < 0.01).

**a**

**c**

**d**

**b**

**Suppl. Figure 5. Infection of Non-Human Vero E6 Cells with Omicron Sublineages.** Vero E6 cells were infected with indicated SARS-CoV-2 viruses: D614G, Delta (B.1.617.2) and selected Omicron sublineages (BA.2, BA.5.1, BQ.1.1, XBB.1.5, XBB.1.9.1 or XBB.1.9.2) at MOI 0.1. Analyses were performed in two independent experiments in technical duplicates. **a** Progeny viruses were collected from supernatant at indicated time points and titrated using standard plaque assay on Vero E6 cells. Replication analysis is shown as mean ± SEM. **b** Heatmap represents viral titers from replication analysis (a) at corresponding time points. **c** Increase of viral titers in early phase of infection was calculated from linear regression between the two initial data points (0 and 16 h p.i.) from replication analysis (a). **d** Area under the curves (AUCs) were calculated from replication analysis in (a). Data are shown as mean ± SEM. Data are shown as boxplots (min to max; box extends from the 25th to the 75th percentile, center line represents median). Statistical analyses were performed using non-paired, non-parametric Kruskal-Wallis test, statistical significances are displayed compared to SARS-CoV-2 D614G (*), BA.2 (Δ) or XBB.1.5 (O) (*p < 0.05; **p < 0.01; ***p < 0.001).

**Table S1.** Characteristics of COVID-19 cases notified to the German national surveillance system through public health authorities and COVID-19 cases with randomly selected diagnostic samples and linked to SARS-CoV-2 full genome sequences, Germany, December 1st, 2021, to April 30th, 2023. Cases for which epidemiological data were not available are categorized as missing and are presented in italics.

|  | **Characteristic** | **COVID-19 cases notified to German national surveillance system** | | **COVID-19 cases with full genome sequences and plausible epidemiological data** | |
| --- | --- | --- | --- | --- | --- |
|  |  | Number | % | Number | % |
|  | **Total** | 32468122 | 100 | 272770 | 0.8 |
|  | **Sex^a^** |  |  |  |  |
|  | Female | 16891676 | 52.0 | 144765 | 53.1 |
|  | Male | 15299244 | 47.1 | 127082 | 46.6 |
|  | *Missing* | *277202* | *0.9* | *923* | *0.3* |
|  | **Age groups (years)** |  |  |  |  |
|  | 0–4 | 861707 | 2.7 | 4393 | 1.6 |
|  | 5–14 | 3808849 | 11.7 | 22538 | 8.3 |
|  | 15–34 | 9423171 | 29.0 | 78241 | 28.7 |
|  | 35–59 | 12760500 | 39.3 | 109590 | 40.2 |
|  | 60–79 | 4323041 | 13.3 | 41290 | 15.1 |
|  | ≥ 80 | 1262350 | 3.9 | 16651 | 6.1 |
|  | *Missing* | *28504* | *0.1* | *67* | *0.02* |
|  | **Hospitalization** |  |  |  |  |
|  | Yes | 598026 | 1.8 | 9182 | 3.4 |
|  | No | 13687958 | 42.2 | 105161 | 38.5 |
|  | *Missing* | *18182138* | *56.0* | *158427* | *58.1* |
|  | **Intensive Care Unit** |  |  |  |  |
|  |  | n/N* | % | n/N* | % |
|  | Yes | 36161/598026 | 6.0 | 828/9182 | 9.0 |
|  | No | 534766/598026 | 89.4 | 8014/9182 | 87.3 |
|  | *Missing* | *27099/598026* | *4.5* | *340/9182* | *3.7* |
|  | **Deaths** |  |  |  |  |
|  | Yes | 65036 | 0.2 | 1285 | 0.5 |
|  | No | 30856636 | 95.0 | 259930 | 95.3 |
|  | *Missing* | *1546450* | *4.8* | *11555* | *4.2* |
|  | **Vaccination** |  |  |  |  |
|  | Yes | 5681654 | 17.5 | 58913 | 21.6 |
|  | No | 1881425 | 5.8 | 16219 | 5.9 |
|  | *Missing* | *24905043* | *76.7* | *197638* | *72.5* |

^a^ Sex at birth.

* n = COVID-19 cases in intensive care unit, N = number of hospitalized COVID-19 cases

**Table S2.** COVID-19 cases with hospitalization among age groups after infection with SARS-CoV-2 Omicron BA.1, BA.2, BA.5.1, BQ.1.1 and with recombinant lineages XBB.1.5, XBB.1.9.1, and XBB.1.9.2, Germany, December 1st, 2021, to April 30th, 2023 (n=1,995). Hospitalized cases for which information on age was not available were categorized as missing and are presented in italics.

| **Hospitalization** | **BA.1** | | | **BA.2** | | **BA.5.1** | | **BQ.1.1** | | **XBB.1.5** | | **XBB.1.9.1** | | **XBB.1.9.2** | | **Total** | |
| --- | --- | --- | --- | --- | --- | --- | --- | --- | --- | --- | --- | --- | --- | --- | --- | --- | --- |
|  | n/N* | | % | n/N* | % | n/N* | % | n/N* | % | n/N* | % | n/N* | % | n/N* | % | n/N* | % |
| **Total** | 99/6825 | | 1.5 | 818/56728 | 1.4 | 398/13058 | 3.0 | 371/5394 | 6.9 | 255/2199 | 11.6 | 26/235 | 11.1 | 28/200 | 14.0 | 1995/84639 | 2.4 |
| **Age groups (years)** | |  | |  |  |  |  |  |  |  |  |  |  |  |  |  |  |
| 0–4 | 1/151 | | 0.7 | 12/1129 | 1.1 | 7/131 | 5.3 | 1/17 | 5.9 | 1/12 | 8.3 | 1/2 | 50.0 | 1/2 | 50.0 | 24/1444 | 1.7 |
| 5–14 | 1/989 | | 0.1 | 16/5524 | 0.3 | 3/501 | 0.6 | 0/94 | 0 | 1/27 | 3.7 | 0/2 | 0 | 0/2 | 0 | 21/7139 | 0.3 |
| 15–34 | 16/2411 | | 0.7 | 68/17019 | 0.4 | 18/3641 | 0.5 | 16/1373 | 1.2 | 7/585 | 1.2 | 2/52 | 3.8 | 2/52 | 3.8 | 129/25133 | 0.5 |
| 35–59 | 16/2486 | | 0.6 | 107/22675 | 0.5 | 48/5508 | 0.9 | 51/2414 | 2.1 | 30/860 | 3.5 | 4/99 | 4.0 | 3/85 | 3.5 | 259/34127 | 0.8 |
| 60–79 | 31/585 | | 5.3 | 273/7733 | 3.5 | 130/2423 | 5.4 | 118/984 | 12.0 | 84/411 | 20.4 | 7/47 | 14.9 | 5/31 | 16.1 | 648/12214 | 5.3 |
| ≥ 80 | 34/203 | | 16.7 | 342/2633 | 13.0 | 192/854 | 22.5 | 185/511 | 36.2 | 132/304 | 43.4 | 12/33 | 36.4 | 17/28 | 60.7 | 914/4566 | 20.0 |
| *Missing* | *0/0* | | *0* | *0/15* | *0* | *0/0* | *0* | *0/1* | *0* | *0/0* | *0* | *0/0* | *0* | *0/0* | *0* | *0/16* | *0* |

* n = number of hospitalizations, N = number of infected cases reported to the German notification system

**Table S3.** Characteristics of SARS-CoV-2 cases included in the sub-analysis on hospitalization, by SARS-CoV-2 lineages BA.1, BA.2, BA.5.1, BQ.1.1, XBB.1.5, XBB.1.9.1 and XBB.1.9.2, Germany, December 1st, 2021, to April 30th, 2023 (n=33,632).

| **Characteristic** | **BA.1** | | **BA.2** | | **BA.5.1** | | **BQ.1.1** | | **XBB.1.5** | | **XBB.1.9.1** | | **XBB.1.9.2** | | **Total** | |
| --- | --- | --- | --- | --- | --- | --- | --- | --- | --- | --- | --- | --- | --- | --- | --- | --- |
|  | Number | % | Number | % | Number | % | Number | % | Number | % | Number | % | Number | % | Number | % |
| **Sub-analysis for**  **hospitalization** | 3188 | 9.5 | 22258 | 66.2 | 5085 | 15.1 | 2042 | 6.1 | 887 | 2.6 | 84 | 0.2 | 88 | 0.3 | 33632 | 100.0 |
| **Sex^a^** |  |  |  |  |  |  |  |  |  |  |  |  |  |  |  |  |
| Female | 1630 | 51.1 | 11942 | 53.7 | 2677 | 52.6 | 1117 | 54.7 | 476 | 53.7 | 50 | 59.5 | 43 | 48.9 | 17935 | 53.3 |
| Male | 1558 | 48.9 | 10316 | 46.3 | 2408 | 47.4 | 925 | 45.3 | 411 | 46.3 | 34 | 40.5 | 45 | 51.1 | 15697 | 46.7 |
| **Age groups (years)** |  |  |  |  |  |  |  |  |  |  |  |  |  |  |  |  |
| 0–4 | 64 | 2.0 | 415 | 1.9 | 45 | 0.9 | 4 | 0.2 | 7 | 0.8 | 1 | 1.2 | 2 | 2.3 | 538 | 1.6 |
| 5–14 | 425 | 13.3 | 2110 | 9.5 | 154 | 3.0 | 42 | 2.1 | 8 | 0.9 | 0 | 0 | 0 | 0 | 2739 | 8.1 |
| 15–34 | 1139 | 35.7 | 6531 | 29.3 | 1445 | 28.4 | 470 | 23.0 | 218 | 24.6 | 22 | 26.2 | 15 | 17.0 | 9840 | 29.3 |
| 35–59 | 1175 | 36.9 | 8745 | 39.3 | 2059 | 40.5 | 824 | 40.4 | 284 | 32.0 | 29 | 34.5 | 33 | 37.5 | 13149 | 39.1 |
| 60–79 | 276 | 8.7 | 3168 | 14.2 | 981 | 19.3 | 419 | 20.5 | 180 | 20.3 | 15 | 17.9 | 15 | 17.0 | 5054 | 15.0 |
| ≥ 80 | 109 | 3.4 | 1289 | 5.8 | 401 | 7.9 | 283 | 13.9 | 190 | 21.4 | 17 | 20.2 | 23 | 26.1 | 2312 | 6.9 |
| **Hospitalization** |  |  |  |  |  |  |  |  |  |  |  |  |  |  |  |  |
| Yes | 99 | 3.1 | 818 | 3.7 | 398 | 7.8 | 371 | 18.2 | 255 | 28.7 | 26 | 31.0 | 28 | 31.8 | 1995 | 5.9 |
| No | 3089 | 96.9 | 21440 | 96.3 | 4687 | 92.2 | 1671 | 81.8 | 632 | 71.3 | 58 | 69.0 | 60 | 68.2 | 31637 | 94.1 |
| **Hospitalization by age groups (years)** |  |  |  |  |  |  |  |  |  |  |  |  |  |  |  |  |
|  | n/N* | % | n/N* | % | n/N* | % | n/N* | % | n/N* | % | n/N* | % | n/N* | % | n/N* | % |
| 0–4 | 1/64 | 1.6 | 12/415 | 2.9 | 7/45 | 15.6 | 1/4 | 25.0 | 1/7 | 14.3 | 1/1 | 100.0 | 1/2 | 50.0 | 24/538 | 4.5 |
| 5–14 | 1/425 | 0.2 | 16/2110 | 0.8 | 3/154 | 1.9 | 0/42 | 0 | 1/8 | 12.5 | 0/0 | 0 | 0/0 | 0 | 21/2739 | 0.8 |
| 15–34 | 16/1139 | 1.4 | 68/6531 | 1.0 | 18/1445 | 1.2 | 16/470 | 3.4 | 7/218 | 3.2 | 2/22 | 9.1 | 2/15 | 13.3 | 129/9840 | 1.3 |
| 35–59 | 16/1175 | 1.4 | 107/8745 | 1.2 | 48/2059 | 2.3 | 51/824 | 6.2 | 30/284 | 10.6 | 4/29 | 13.8 | 3/33 | 9.1 | 259/13149 | 2.0 |
| 60–79 | 31/276 | 11.2 | 273/3168 | 8.6 | 130/981 | 13.3 | 118/419 | 28.2 | 84/180 | 46.7 | 7/15 | 46.7 | 5/15 | 33.3 | 648/5054 | 12.8 |
| ≥ 80 | 34/109 | 31.2 | 342/1289 | 26.5 | 192/401 | 47.9 | 185/283 | 65.4 | 132/190 | 69.5 | 12/17 | 70.6 | 17/23 | 73.9 | 914/2312 | 39.5 |

* n = number of hospitalizations, N = number of infected cases reported to the German notification system
